# Molecular roles of microRNA-21 and exosomal miR-21 in gastrointestinal cancers: diagnostic, therapeutic, and drug resistance insights

**DOI:** 10.3389/fmolb.2025.1697875

**Published:** 2025-12-16

**Authors:** Chunguang Wang, Meiling Bai, Xingliang Liu, Zhijun Li, Haibin Wang, Shengbin Guo

**Affiliations:** 1 The First Affiliated Hospital of Hebei North University, Zhang Jiakou, Hebei, China; 2 Basic Medical College of Hebei North University, Zhang Jiakou, Hebei, China; 3 The Second Hospital of Zhangjiakou, Zhang Jiakou, Hebei, China; 4 Zhangjiakou University, Zhang Jiakou, Hebei, China

**Keywords:** gastrointestinal cancers, microRNA-21, exosomes, diagnostics, overcoming drug resistance

## Abstract

MicroRNA-21 (miR-21) and its exosomal variant have gained recognition as pivotal molecular contributors to the etiology and advancement of gastrointestinal (GI) neoplasms, encompassing colorectal, gastric, pancreatic, and esophageal cancers. From a biosciences standpoint, miR-21 operates as a formidable oncomiR by inhibiting tumor suppressor genes, consequently fostering the dysregulated activation of crucial signaling cascades. The exosomal form of miR-21, released through tumor-derived extracellular vesicles, enhances intercellular interactions within the tumor microenvironment, influencing processes such as angiogenesis, immune evasion, epithelial-mesenchymal transition (EMT), and metastasis. Clinically, both tissue and circulating (serum/plasma) concentrations of miR-21 exhibit substantial potential as non-invasive biomarkers for the early detection, disease stratification, and prognostic assessment in gastrointestinal malignancies. Increased levels of exosomal miR-21 are associated with diminished overall survival, lymph node dissemination, and resistance to chemotherapeutic agents such as 5-fluorouracil, cisplatin, and gemcitabine. Mechanistically, exosomal miR-21 facilitates drug resistance by inhibiting apoptotic pathways and promoting cellular longevity through the modulation of the tumor microenvironment and stromal-tumor interactions. Therapeutically, bioscience-oriented strategies aimed at targeting miR-21 are currently under scrutiny to counteract chemoresistance and restore therapeutic effectiveness. These methodologies possess significant potential for applications in personalized medicine concerning gastrointestinal cancers. This review synthesizes contemporary biosciences perspectives on the molecular roles of miR-21 and exosomal miR-21, underscoring their diagnostic, prognostic, and therapeutic significance in gastrointestinal neoplasms. Particular emphasis is directed toward their involvement in overcoming drug resistance, thereby establishing them as promising targets for forthcoming translational oncology investigations.

## Introduction

MicroRNAs (miRNAs) are small non-coding RNAs that play crucial roles in post-transcriptional gene regulation. They typically target the 3′-untranslated regions (3′-UTRs) of messenger RNAs (mRNAs), leading to mRNA degradation or translational repression for approximately 60% of all coding genes (Dhawan et al., 2018). In complex organisms, miRNAs are essential for “fine-tuning” intricate cellular processes, including proliferation, differentiation, and migration (Nagel et al., 2016). Furthermore, miRNAs are key regulators of immunological responses, inflammation, and wound healing processes (Huynh et al., 2019).

Dysregulation of miRNA expression is a common feature in many human diseases, including cancer. Tumors often exploit miRNA-dependent biological mechanisms involved in processes like inflammation and wound healing to drive their own progression (El-Mahdy et al., 2022). Specific miRNAs that promote tumorigenesis are termed “oncomiRs.” Panels of miRNAs have shown potential as diagnostic or prognostic biomarkers across a wide range of malignancies, including breast (Elrebehy et al., 2023), gastric (Zheng et al., 2013), thyroid (Doghish et al., 2023), colorectal (Fathi et al., 2023), kidney (Canatan et al., 2021), and lung cancers (Ahadi, 2021).

OncomiRs function by targeting and inhibiting tumor suppressor (TS) genes, thereby promoting cancer cell survival, proliferation, and metastasis. Common TS genes targeted by various oncomiRs include *PTEN*, Reversion-inducing cysteine-rich protein with Kazal motifs (*RECK*), Tropomyosin 1 (*TMP1*), and Programmed Cell Death 4 (*PDCD4*) (Park et al., 2021).

**FIGURE 1:**
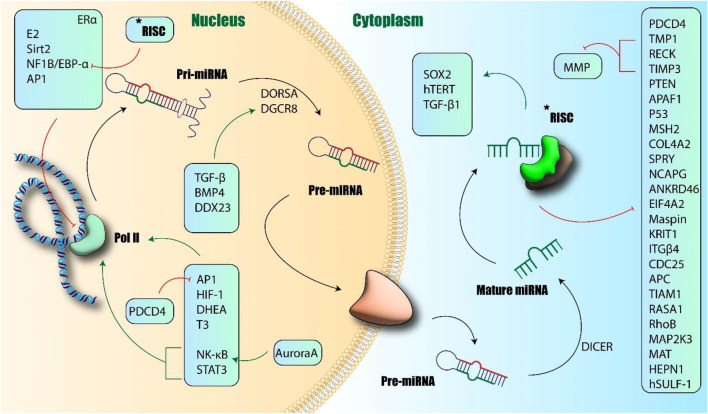
Biogenesis and function of miR-21. Induction of miR-21 transcription occurred via Pol II and further production of Pri-miRNA-21. Different transcriptional factors could induce or inhibit this process. Finally, mature miR-21 changes the transcription of other proteins and induces or inhibits their expression. APAF1: Apoptotic Protease Activating Factor 1; ANKRD46: Ankyrin Repeat Domain 46; APC: Adenomatous Polyposis Coli; AP1: Activator Protein 1; BMP4: Bone Morphogenetic Protein 4; CDC25: Cell Division Cycle 25; COL4A2: Collagen Type IV Alpha 2; DDX23: DEAD-Box Helicase 23; DICER: Dicer Ribonucléase; DHEA: Dehydroepiandrosterone; DGCR8: DiGeorge Syndrome Critical Region Gene 8; DROSHA: Dicer-Related RNA Helicase; EIF4A2: Eukaryotic Translation Initiation Factor 4A2; ER-alpha: Estrogen Receptor Alpha; HEPn: Hepatocellular Carcinoma Expressed Protein; HIF-1: Hypoxia-Inducible Factor 1; ITGbeta: Integrin Beta; KRIT1: Krit1 Rho GTPase Activating Protein; MAPK: Mitogen-Activated Protein Kinase; MAT: Methionine Adenosyltransferase; MSH: MutS Homolog; NF1B: Neurofibromin 1; NK-κb: Nuclear Factor kappa B; PDCD4: Programmed Cell Death 4; PTEN: Phosphatase and Tensin Homolog; RASA1: Ras p21 Protein Activator 1; RECK1: Reversion-Inducing Cysteine-Rich Protein with Kazal Motifs 1; RhoB: Ras Homolog Family Member B; SOX2: SRY-Box Transcription Factor 2; SPRY: Sprouty; STAT3: Signal Transducer and Activator of Transcription 3; TGF-b: Transforming Growth Factor Beta; TGF-B: Transforming Growth Factor Beta; TIMP3: Tissue Inhibitor of Metalloproteinase 3; TIAM1: T-Cell Immunoglobulin and Mucin-Domain Containing Protein 1 (author-created).

Among the most significant and widely studied oncomiRs across the majority of human malignancies is microRNA-21 (miR-21) (Figure 1) (Park et al., 2021). It is noteworthy that most functional studies in this context focus on the miR-21-5p strand as the primary mature, active product, and detailed comparative mechanistic data for the miR-21-3p strand in GI cancers remain limited in the current literature.

A central mechanism underlying miR-21’s oncogenic activity in numerous cancers involves its regulation of the Phosphatase and Tensin Homolog (*PTEN*)/Phosphoinositide 3-kinase (PI3K)/Protein Kinase B (Akt) signaling pathway. MiR-21 directly binds to the 3′-UTR of *PTEN* mRNA, leading to translational repression or mRNA degradation, thereby reducing the levels of the PTEN tumor suppressor protein (Zhu et al., 2008; Khan et al., 2016; Meng et al., 2007). Since PTEN normally antagonizes PI3K signaling by dephosphorylating PIP3, its downregulation by miR-21 results in the hyperactivation of PI3K and its downstream effector Akt. Activated Akt promotes cell survival (by inhibiting apoptosis), proliferation, cell growth (partly through mechanistic Target Of Rapamycin (mTOR) signaling), migration, and invasion, and contributes significantly to chemoresistance (Song WF. et al., 2013; Vahidnezhad et al., 2016; Li et al., 2014). This miR-21/*PTEN*/PI3K/Akt axis represents a core oncogenic signaling node frequently exploited in GI malignancies.

High levels of miR-21 expression are frequently observed in gastric, colorectal, lung, pancreatic, breast, and ovarian cancers, among others (Zhu et al., 2008; Wang et al., 2021a; Huang et al., 2021; Jiang et al., 2021; Feng and Tsao, 2016). Clinically, elevated miR-21 expression often correlates with a worse patient prognosis, increased lymph node metastasis (Arisan et al., 2021; Asangani et al., 2008), and the development of chemoresistance (Petrocca et al., 2008). Functional studies support its oncogenic role; induced miR-21 expression can cause malignant B-cell lymphoma in preclinical models, while its genetic silencing reduces carcinogenesis in transgenic mice (Rihane et al., 2022).

It is important to acknowledge, however, that while the vast majority of evidence points to miR-21 acting as a potent oncomiR in GI malignancies, isolated studies have reported potential anti-tumorigenic effects in specific contexts, such as certain models of colon and liver cancer (Xu et al., 2012; Wang et al., 2014). These seemingly contradictory findings underscore the complexity of miRNA function. Such discrepancies might arise from several factors. Cellular and tissue context is paramount; the downstream consequences of miR-21 targeting specific genes (like PTEN or PDCD4) may differ significantly depending on the specific GI cell type, the cancer stage, and the surrounding tumor microenvironment (TME). Furthermore, variations in experimental model systems–ranging from simplified *in vitro* cultures lacking TME interactions to different types of *in vivo* animal models (e.g., genetic vs. xenograft) and analyses of heterogeneous human tumor samples–can yield divergent results. Methodological differences, such as the techniques used to modulate miR-21 levels (e.g., mimics vs. inhibitors, transient vs. stable expression) and the specific functional assays employed, can also contribute to variability. Finally, given that miR-21 regulates a complex network of potentially hundreds of target mRNAs, its ultimate biological effect likely represents the net outcome of modulating multiple pathways simultaneously, which could feasibly shift between pro- and anti-tumorigenic depending on the specific cellular state and environmental cues. Understanding these context-dependent factors is crucial for accurately interpreting miR-21’s role and harnessing its therapeutic potential.

This review summarizes the function of miR-21, and particularly its exosomal form, in GI cancers. The literature search for this review was conducted using PubMed, Scopus, Web of Science, and Google Scholar databases, covering articles published up to September 2024. Keywords included “miR-21,” “exosomal miR-21,” combined with “esophageal cancer,” “gastric cancer,” “pancreatic cancer,” and “colorectal cancer.” Only English language articles were included.

It is important to acknowledge, however, that while the vast majority of evidence points to miR-21 acting as a potent oncomiR in GI malignancies, isolated studies have reported potential anti-tumorigenic effects in specific contexts, such as certain models of colon and liver cancer (26, 27). These seemingly contradictory findings underscore the complexity of miRNA function. Such discrepancies might arise from several factors. Cellular and tissue context is paramount; the downstream consequences of miR-21 targeting specific genes (like PTEN or PDCD4) may differ significantly depending on the specific GI cell type, the cancer stage, and the surrounding tumor microenvironment (TME). Furthermore, variations in experimental model systems–ranging from simplified *in vitro* cultures lacking TME interactions to different types of *in vivo* animal models (e.g., genetic vs. xenograft) and analyses of heterogeneous human tumor samples–can yield divergent results. Methodological differences, such as the techniques used to modulate miR-21 levels (e.g., mimics vs. inhibitors, transient vs. stable expression) and the specific functional assays employed, can also contribute to variability. Finally, given that miR-21 regulates a complex network of potentially hundreds of target mRNAs, its ultimate biological effect likely represents the net outcome of modulating multiple pathways simultaneously, which could feasibly shift between pro- and anti-tumorigenic depending on the specific cellular state and environmental cues. Understanding these context-dependent factors is crucial for accurately interpreting miR-21’s role and harnessing its therapeutic potential.

## MicroRNAs and gastrointestinal cancer

### MiR-21 and pancreatic cancer

Cancer stem cells, also known as CSCs, have been linked to chemoresistance and are crucial in the development of various cancers, such as pancreatic ductal adenocarcinoma (PDAC) (Luo et al., 2020). High autophagic flux in CSCs produces stress in the microenvironment, which in turn increases ATP-binding cassette (ABC) transporter expression, multidrug resistance genes, and anti-apoptotic proteins (An and Yang, 2020). Pancreatic Cancer Stem Cells (PCSCs), which make up just under one percent of all pancreatic cancer cells, are rare and they promote the growth, preservation, spread, and treatment resistance of PDAC tumors (Vandewalle et al., 2021). Epithelial-mesenchymal transition (EMT) is crucial for cancer cell invasion and metastasis. This process involves increased cell mobility, reduced cell adhesion, the repression of epithelial markers like E-cadherin, and the upregulation of mesenchymal markers including N-cadherin, Vimentin, and transcription factors like *Snail* and Zinc Finger E-Box Binding Homeobox 1 (*ZEB1*) (Wang et al., 2021b). In PDAC, dysregulation of E-cadherin correlates with a worse prognosis, poorer differentiation, and therapy resistance (Medina et al., 2010).

Key regulators of EMT are often overexpressed in PDAC and linked to aggressive phenotypes. For example, *Snail* expression is elevated in ∼80% of PDAC patients, correlating with higher tumor grade and lymph node invasion (Li et al., 2009). Similarly, *ZEB1* overexpression, potentially driven by NF-κB activation, is linked to aggressiveness and poor prognosis (Correia de Sousa et al., 2021). *ZEB1* also suppresses miR-200 family members and miR-203, which normally inhibit stemness factors and *ZEB1* itself, creating complex feedback loops (Jiang et al., 2020). Vimentin overexpression also promotes PDAC spread, reduces survival, and is linked to gemcitabine resistance (Kim et al., 2011; Klonisch et al., 2008). Non-canonical Wnt-11 expression is another factor associated with advanced staging and poor prognosis (Li et al., 2007). The complex regulation of EMT involves multiple layers, including alternative splicing, post-translational modifications, non-coding RNAs, and chromatin remodeling (Niess et al., 2015). Notably, PDAC cells undergoing EMT often acquire cancer stem cell (CSC) traits, highlighting a close relationship between these two processes (Miranda-Lorenzo et al., 2014).

CD133, CD44, Lgr5, ESA/EpCAM (epithelial-specific antigen), CD24, CXCR4, aldehyde dehydrogenase-1 (ALDH1), and DclK1 are the primary markers of PCSCs. Other markers include (Valle et al., 2018). It's important to note that pathways involving EMT and autophagy are directly related to indications of CSCs as PDAC progresses (Marcato et al., 2011). Pancreatic ductal adenocarcinoma patients with the expression of cMet+ CD45^+^ CD34^+^CD133 Ter119, CD44, Pdx1, CD13, CD9, CD133, and CD24 had a poor prognosis (PDAC). Recent research has shown that advanced pancreatic intraepithelial neoplasia is associated with more CD44, CD24, CXCR4, ESA, and nestin levels (PanIN) (Santamaria et al., 2017).

#### MiR-21 regulation of cancer stem cells (CSCs) and EMT

MiR-21 is strongly expressed in CSCs, and its significance in PDAC was studied by Mortoglou et al. *in vitro*. After miR-21 was removed from the human PDAC cell lines Panc-1 and MiaPaCa-2 using a knockout (KO) technique, reversible manifestations of epithelial-mesenchymal transition (EMT) along with cancer stem cells’ signs were found. Depending on miR-21 expression levels, the expression patterns of important CSC markers as CD44, CX-C chemokine receptor type 4 (CXCR4), CD133, and ALDH1 vary. These markers include the CX-C chemokine receptor type 4, CD44, and CD133, as well as ALDH1. Using standard cell viability and invasion assays, Panc-1 and MiaPaCa-2 cell growth as well as their invasive potential were both reduced by miR-21 KO. These findings imply that miR-21 expression correlates with PDAC aggressiveness and may serve as a potential biomarker (Bao et al., 2012; Zhao et al., 2018). Beyond intrinsic cell properties, miR-21 also influences the tumor microenvironment. The PDAC tumor microenvironment has an abundance of immunosuppressive M2 macrophages that serve as a home for parental PDAC cells to proliferate, colonize, and re-generate (Dawood et al., 2014). M2 macrophages are lethal to PDAC patients and reduce the effectiveness of treatment (Lei et al., 2017). M2 macrophage infiltration is also to blame for the paradoxical survival of PDAC cases (Cojoc et al., 2015). By delivering genes into the tumor microenvironment, exosomes encourage tumor progression (Dalla Pozza et al., 2015). Exosomes made by M2 macrophages promote the growth of PDAC cells, which in turn causes the tumor (Kalluri and Weinberg, 2009). Exosomes themselves and exosomal microRNA (miR)-501-3p, which is created by M2 macrophages, are both strong agents that can cause PDAC cells to invade, migrate, spread, and form tumors (Felipe Lima et al., 2016). More specifically, Exosomes produced by M2 macrophages have higher miR-21-5p levels, recommending that such particles might have a role in regulating migration and invasion of colorectal cancer cells (Lamouille et al., 2014). Transcriptional factors Krüppel-like factors (KLFs) are important in PDAC. It was discovered that the miR-21 target gene KLF3 controls a subset of KLFs (Iacobuzio-Donahue et al., 2009). Interestingly, it has a connection to esophageal squamous cell cancer’s stem cell-like features (Winter et al., 2008). To add, KLF3 may help with prostate cancer prognosis (Renz et al., 2017).

The study of Chang et al. sought to understand how the miR-21-5p necessary for PDAC stem cell activity and differentiation is carried by M2 macrophage-produced Extracellular Vesicles (EVs), which aid in the development of PDAC. Polarized M2 macrophages were used to extract the EVs. EVs generated from M2 macrophages were examined for miR-21-5p. CD24+CD44+EpCAM+ *in vitro* co-cultures of M2 macrophages with either high or low miR-21-5p levels and stem cells were carried out. Using assays like qPCR/Western blot for Oct4/Nanog, sphere/colony formation assays, transwell migration/invasion assays, apoptosis assays (e.g., Annexin V staining), and mouse xenograft models for tumorigenicity, the effects of EVs and miR-21-5p were examined. KLF3 expression and miR-21-5p activity were investigated. The increased PDAC stem cell activity is believed to be caused by the EVs that M2 macrophages create. In M2 EVs, miR-21a-5p levels were elevated. PDAC stem cells’ capacities for proliferation, differentiation, colony formation, invasion, migration, and anti-apoptosis were suppressed *in vitro* and *in vivo* when expression of Nanog/Oct4 within M2 macrophage-derived EVs has been reduced. In PDAC stem cells, it was obvious that miR-21-5p controlled KLF3 expression and activity. Exosomal miR-21a-5p from M2 macrophages was found to increase PDAC stem cell activity and proliferation while suppressing PDAC stemness by targeting KLF3 (Arumugam et al., 2009).

Other enzymatic pathways within cancer cells are also modulated by miR-21 activity and related inhibitors.

#### Modulation by peptidylarginine deiminase (PAD) inhibitors

A class of calcium-dependent enzymes known as peptidylarginine deiminases (PADs) modify target proteins’ structure and function post-translationally by, among other things, having an impact on protein-protein interactions, altering gene regulation and producing new epitopes (Buck et al., 2007). This post-translational change may facilitate protein moonlighting, a phylogenetically evolved mechanism that allows molecules to carry out many roles on the inside of a single polypeptide chain, in terms of both physiological and pathological features (Maier et al., 2010). Five isozyme-specific PADs (Uysal-Onganer et al., 2010), there are known mammalian transcription factors, each having a distinctive affinity for protein targets and pattern of expression. Targeting specific PAD isozymes for different cancer types and subtypes is an active area of research, focusing on the relative significance of the three main isozymes (PAD2, PAD3, and PAD4) in malignancies (Arisan et al., 2020).

In a study by Uysal-Onganer et al., MiaPaCa-2 PDAC and Panc-1 cell lines were handled by the isozyme-specific inhibitors of phosphodiesterase-2 (PAD2), −3, and −4 (PAD2, PAD3, and PAD4) as well as the pan-PAD inhibitor Cl-amidine. The implications for moesin, mitochondrial housekeeping (prohibitin, PHB), and gene regulation were studied in relation to the effects on cellular protein expression. Modifications in EV signatures were also examined (deiminated histone H3, citH3). It was determined that Panc-1 cells expressed PAD2 and PAD3 at higher levels than MiaPaCa-2 cells, indicating that Panc-1 and PAD3 were both identified as pancreatic cancer cell lines. The moesin expression, a protein which has been dysregulated in pancreatic cancer and is related to the severity of the illness, rose in response to therapy with a PAD2 isozyme-specific inhibitor, which proved to be most effective in preventing the invasion of Panc-1 cells. PHB levels decreased as well, albeit the change was not statistically significant, and the results for histone H3 deamination were mixed. Treatment with a PAD inhibitor altered EV signatures, with miR-21 and miR-221, two pro-oncogenic microRNAs, and miR-155, an anti-oncogenic microRNA, being significantly reduced and miR-155 increased, respectively (miR-126). However, blocking PAD4 had no appreciable effect, and blocking all PADs with Cl-amidine had even less of an impact. Limiting PAD4 had no noticeable impact, whereas inhibiting PAD2 and PAD3 had the greatest impact on reducing moesin expression, avoiding cancer cell invasion, and changing EV markers. It is noteworthy that Panc-1 cells also responded strongly to PAD3 inhibitor, validating previous results that Panc-1 cells exhibit more neuronal/stem-like properties than MiaPaCa-2 cells. Their results disclose new regulatory functions for PAD isozymes in PDAC and emphasize the need of tailoring PAD isozyme therapy to the individual disease and cancer subtype being treated (Guo and Wang, 2009). Furthermore, miR-21 interacts with key tumor suppressor pathways sensitive to the hypoxic tumor environment.

#### Targeting the VHL/HIF-1α axis

VHL is a ubiquitin ligase E3 component of elongin B, elongin C, and cullin-2. When oxygen is plentiful, prolyl hydroxylase proteins hydroxylate hypoxia-inducible factor (HIF)-1, that VHL subsequently ubiquitinates and degrades (Wang L. et al., 2018). Many solid tumors are anoxic, and VHL inactivation may upregulate HIF-1, encouraging tumor growth. VHL might be suppressed in malignancies even though it typically suppresses tumors through mechanisms including microRNA regulation. MiR-101 and (Wang et al., 2016a) miR-155 influence VHL expression (Hotz et al., 2007).

It has been demonstrated that miR-21’s deleterious impact on VHL, its target, is real. MiR-21 inhibition reduced Matrix Metalloproteinase (MMP)-9 and MMP-2 expression plus the HIF-1/Vascular Endothelial Growth Factor (VEGF) pathway. MiR-21 silencing inhibited tumor development *in vivo*. MiR-21 knockdown reduced pancreatic cancer infiltration, motility, and growth through inhibiting the HIF-1/VEGF pathway, MMP-2, and MMP-9 expression. Thus, miR-21/VHL could be used as a viable novel pancreatic cancer therapeutic and preventative target (De Craene and Berx, 2013). Finally, miR-21’s influence extends to stromal components and therapy response.

#### Role in cancer-associated fibroblasts (CAFs) and chemoresistance

Several research have demonstrated that miR-21 is crucial for regulating the activation of transforming growth factor beta (TGF) in fibroblasts (Fedele et al., 2017). However, it is unclear if miR-21 in CAFs might change the microenvironment of the PDAC tumor and promote treatment resistance. Using a tumor sample from a patient who had been diagnosed with PDAC, Zhang et al. investigated the connection among CAF activation, miR-21 transcription, and treatment tolerance. They experimented with manipulating miR-21 expression in CAFs to see how it affected CAF function regulation. Gemcitabine-resistant pancreatic cancer patients have been identified as having greater levels of miR-21 expression along with greatly activated CAFs. Strong miR-21 expression in CAFs was found to increase PDAC cell line penetration, MMP-3 and MMP-9 levels and Chemokine Ligand (CCL)-7 as well as Platelet-Derived Growth Factor (PDGF), in an *in vitro* investigation. There *in vivo* study found that miR-21 downregulation in CAFs decreased desmoplasia and improved the gemcitabine’s benefits, but PDAC desmoplasia was worsened by miR-21 overexpression in CAFs, and the condition was more difficult to cure with gemcitabine. They concluded that miR-21 encouraged CAF activation, that influenced PDAC’s treatment resistance (Klymkowsky and Savagner, 2009). Table 1 lists various researches on miR-21 and pancreatic cancer.

**TABLE 1:** miR-21 and pancreatic cancer.

Status of expression	Target	Model	Cell line type	Effect	Conclusion	Ref.
Up	Snail, ZEB1, Wnt-11, CD24, CD44, CD133, CD13, ALDH1, CXCR4	*In vitro*	Panc-1, MiaPaCa-2, BxPC-3, HPDE; H6c7)	Enhanced the expression of biomarkers related to the cancer stemness and progression	Increase of cancer progression and invasion	Plaks et al. (2015)
Up	Spry2, MAPK, PI3K	Human serum *In vitro*, *In* *vivo*	PANC-1, MIA PaCa-2, CFPAC-1, HPDE6-c7, SW-1990, AsPC-1	Enhanced the expression of biomarkers related to the cancer stemness and progression	Promoted EGF-induced proliferation, inhibited cell apoptosis and accelerated cell cycle progression	Traub et al. (1985)
Up	-	Human serum	-	Increase in pancreatic cancer patients	It could be used as a cancer biomarker for early detection of pancreatic cancer	Yachida and Iacobuzio-Donahue (2009)
Up	-	Human serum	-	Increase in pancreatic cancer patients	It could be used as a cancer biomarker for early detection of pancreatic cancer	Javle et al. (2007) Shibue and Weinberg (2017)
Up	-	Human serum	-	Increase in pancreatic cancer patients	It could be used as a cancer biomarker for early detection of pancreatic cancer	De Craene and Berx (2013)
Up	Mitochondrial pathway	*In vitro*, *In vivo*	Mia PaCa-2 Human PDA-derived Capan-2	Inhibition of apoptosis	Induction of apoptosis by inhibition of miR-21 expression	Rhim et al. (2012)
Up	PTEN	*In vitro*	PSCs, Panc-1	Increase of cancer stemness and progression via induction of PTEN expression	Increase of cancer progression and invasion	Rao and Mohammed (2015)
Up	Slug	*In vitro*	PANC-1	Induction of Slug expression	Increase of cancer invasiveness and metastasis	Chalquest (1986)
Up	SMAD7	Human *In vitro*, *In vivo*	-	It inhibits SMAD7 expression	Increase of cancer invasiveness and metastasis	Hruban et al. (2006)
Up	KLF3	*In vitro*, *In vivo*	PC-3, Capan-1, AsPC-1, PANC-1, HPC-Y5, THP-1	Induction of KLF3 expression	Induction of cancer progression	Arumugam et al. (2009)
Up	-	Human serum	-	Increase in pancreatic cancer patients	It could be used as a cancer biomarker for early detection of pancreatic cancer	Oshima et al. (2007)
Up	PTEN, PDCD4	*In vitro*	PATU8988, PANC-1, 293 TN	Induction of PTEN and PDCD4 expression	Induction of cancer progression and further metastasis risk	Wang et al. (2019a)
Up	-	Human serum	-	Increase in pancreatic cancer patients	It could be used as a cancer biomarker for early detection of pancreatic cancer	Lee et al. (2014)
Up	FasL	Human, *In vitro*, *In vivo*	PANC‐1, BxPC3	Increase of the expression of FasL along with reduction of miR-21 expression following chemotherapy	Chemotherapy resulted in the reduction of miR-21 while induced FasL expression and further inhibited tumor metastasis	Liu et al. (2018a)
Up	PTEN	Human, *In vitro*	HPAC, PANC-1	Enhanced invasion and metastasis, increased miR-21 expression, decreased PTEN, elevated pAKT level were demonstrated in gemcitabine-resistant HPAC and PANC-1 cells	MiR-21 upregulation induced by histone acetylation in the promoter zone is associated with chemoresistance to gemcitabine and enhanced malignant potential in pancreatic cancer cells	Li et al. (2015a)
Up	RECK	Human, *In vitro*	hTERT-HPNE, Hs27, LPc006, LPc028, LPc033, LPc067, LPc111, LPc167, PP437	Pre-miR-21 transfection significantly decreased antiproliferative effects and apoptosis	miR-21 expression correlated with outcome in PDAC patients treated with gemcitabine	Mortoglou et al. (2022)
Up	Bcl-2	*In vitro*	MIA PaCa-2	Upregulation of Bcl-2 expression was detected in cells transfected with miR-21 mimics, accompanied by downregulated Bax expression, less apoptosis, lower caspase-3 activity, decreased chemosensitivity to gemcitabine and increased proliferation compared with the control cells	Upregulation of Bcl-2 directly induced by miR-21 is associated with apoptosis, chemoresistance and proliferation of MIA PaCa-2 pancreatic cancer cells	Chandrakesan et al. (2020)
Up	PDCD4	Human, *In vitro*, *In vivo*	MIA Paca-2, CAFs, Panc02	miR-21 overexpression contributed to the activation of cancer-associated fibroblasts (CAFs) by regulating the PDCD4 gene	miR-21 promoted the activation of CAFs and contributed to the drug resistance of PDAC.	Klymkowsky and Savagner (2009)
Up	CDK6, IRAK3, NRP1, SMAD7 SOCS6, C5ORF41, KLF12, MAPK10, EFNA1, ZBTB41	Human, *In vitro*, *In vivo*	L3.6 pL	The administration of antagomir-21 significantly induced reduction of L3.6 pL cell proliferation, invasion, and chemoresistance against gemcitabine and 5-Fluorouracil	inhibition of miR-21 appear particularly suitable to target stem-like subpopulations and address their specific biological function to promote tumor progression in pancreatic cancer	Hu et al. (2020)
Up	PTEN, p85α	*In vitro*	MiaPaCa-2, PANC-1	Overexpression of miR-21 resulted in decreased levels of p85α and increased phosphorylation of AKT.	miR-21 can influence PI3K-AKT signaling via its direct regulation of p85α	Yin et al. (2020)
Up	PTEN	*In vitro*	HPDE6-C7, SW 1990, CAPAN-1, JF305, PANC-1, BxPC-3	CACS2 overexpression inhibited the migration and invasion of PANC-1 cells by targeting MiR-21	A novel regulatory mechanism of the CASC2/miR-21/PTEN axis that may be important in pancreatic cancer	Yin et al. (2019)
Up	-	Human serum	-	miR-21 could distinguish PC patients from those with other GI cancers or benign pancreatic diseases (BPD)	As new combined microRNA and protein plasmatic biomarker panel for pancreatic cancer	Lan et al. (2019)
Up	-	*In vitro*	AsPC-1, BxPC-3	Hypoxic conditions resulted in direct binding of HIF-1α to the predicted binding site in miR-21	MiR-21 overexpression allows cells to avoid apoptosis in a hypoxic microenvironment	Zhai et al. (2019)
Up	Tgfbr2, Tgfbi, Sox2, Sox5, Sox7, PTEN, TPM1, PDCD4, Maspin, Rasa1and2, Cstf3	Human, *In vivo*	-	The expression of miR-21 was significantly upregulated in human PC tissues as compared to the cancer-adjacent normal tissues	Induction of cancer progression	Liu et al. (2020a)
Up	PDCD4, BTG2	Human, *In vitro*	PANC-1, MiaPaca-2	CRISPR-Cas9 cellular model was generated to knock-out the expression of miR-21 in PANC-1 cells	Importance of miR-148a and miR-21 interactions	Meng et al. (2020)
Up	-	Human tissue	-	Increase in mucinous versus nonmucinous cysts	profiling miRNAs in pancreatic cyst fluid samples is feasible and can yield potential biomarkers for the classification of cystic lesions of the pancreas	Vossenaar et al. (2003)
Up	-	Human, *in vivo*	-	increased when comparing normal tissues, premalignant lesions and invasive carcinoma in the mouse model	Circulating miRs could serve as indicators of drug response	György et al. (2006)
Up	-	Human tissue	-	the expressions of miR-21 and miR-155 were associated with tumor stage and poor prognosis	miR-21 and miR-155 renders necessary the revision of use of microRNAs as biological markers	Wang and Wang (2013)
Up	PDCD4, TIMP3	Human tissue	-	PDCD4 (reduced nuclear staining) and TIMP3 (downregulated expression) and increased miR-21 expression	miR-21 downregulates tumor suppressors PDCD4 and TIMP3, causing tumor growth and a poor clinical fate in pancreatic ductal adenocarcinoma	Henderson and Martin (2014)
Up	BCL-2	*In vitro*	PANC-1, CFPAC-1 MIA Paca-2	MiR-21 downregulation inhibits BCL-2 expression in PANC-1, CFPAC-1, and MIA Paca-2 cells	Resveratrol inhibits miR-21-regulated BCL-2 expression, affecting apoptosis	Jeffery (2018)
Up	-	*In vivo*	-	PFOS exposure positively regulate apoptosis and cell proliferation in cancer	PFOS exposure altered the expression of a suite of miRNAs	Kosgodage et al. (2018)
Up	RECK, PTEN	*In vitro*	PANC-1, MIA PaCa-2, HS766T, SW 1990, PL45, MPanc96, Capan-1	Gemcitabine was sensitized by miR-21 and miR-221, and antisense-gemcitabine combos were synergistic at high fraction impacted	miR-21 antisense oligonucleotides targeting miRNA kill cells under diverse situations and may be a new pancreatic cancer treatment	Uysal-Onganer et al. (2021)
Up	-	Human tissue	-	miR-21 was significantly increased in cancer fine-needle aspirates	feasibility of miRNA profiling on fine-needle aspirated pancreatic cancer specimens	de Paulsen et al. (2001)
Up	-	Human serum/tissue	-	miR-21 is involved in the development of pancreatic cancer critical cancer-associated cellular pathways	miR-21 can be use potential biomarker	Liu et al. (2016a)
Up	-	Human tissue/serum	-	miR-21 was overexpressed by cancer- and juxta-tumoral stromal cells	PTEN silencing by miR-21 in cancer promote tumor growth	Kong et al. (2014)
Up	PTEN	*In vitro*	-	miR-21 family was markedly over-expressed in chemo-resistant PC cell lines	Mir-21 could serve as potential biomarker for tumor aggressiveness	Sun et al. (2019)
Up	PTEN, PDCD4, Maspin, TPM1	Human, *In vitro*	MIAPaCa-2, AsPc-1	borderline significant overexpression of miR-21 was observed in PanIN-3 only	miR-21 abnormalities at the stage of PanIN-3 lesions. and IAP was observed	Yao et al. (2011)
Up	-	Human serum	-	miR-21 was upregulated in 90.6% of the tumors, no associations with outcomes were found	independent prognostic markers in gastrointestinal cancer patients	Li et al. (2013a)
Up	-	Human serum	-	-	Can be use as miRNA prediction model differentiate colorectal neoplasia	Liu et al. (2010a)
Up	-	*In vitro*	CAF-19, COLO-357, MIAPaCa-2, nhPSCs	-	targeting these miRNAs could be useful for developing precision medicine for the prevention of tumor progression	Li et al. (2016a)
Up	-	Human tissue	-	specific microRNAs like miR-21, are differentially expressed between tumor buds and main tumor cells	miR-21 could represent a treatment option for aggressive pancreatic cancer	Zhang et al. (2018a)
Up	-	Human tissue	-	miR-21 demonstrating highest relative fold-changes in the precursor lesions	Overexpression of miR-21 is an early event in the multistage progression of pancreatic cancer	Zhao et al. (2018)
Up	-	Human tissue	-	miR-21 were upregulated	Pancreatic cancer tissue expresses microRNA differently from other periampullary tumors	Yu et al. (2021)
Up	PDCD4	Human, *In vitro*	MIA-Pa-Ca-2, HUP-T3, PSN-1 PDAC	miR-21 levels in the primary tumours correlated with disease stage	miRNA expression profiles may be used as biomarkers for detecting pancreatic cancer	Abue et al. (2015)
Up	PDCD4	*In vitro*	Panc-1	Downregulation of miR-21 by I3C was positively-correlated in a time- and dose-dependent manner	I3C would be effective for enhancing sensitivity of pancreatic cancer cells to gemcitabine via downregulation of miR-21	Škrha et al. (2015)
Up	-	Human saliva	-	hsa-miR-21 being significantly upregulated in saliva of pancreatic cancer patients compared to control	Suggest use of salivary miRNA as biomarker for the early diagnosis of patients with unresectable pancreatic cancer	Yan et al. (2018)
Up	-	Human serum	-	miR-21 levels in serum were significantly associated with overall PaC survival	miRNA-based biomarker can serve as a novel noninvasive approach for PaC diagnosis and prognosis	Liu et al. (2016b)
Up	-	*In vitro*	-	miR-21 was significantly overexpressed in PDAC	These markers may distinguish PDAC and its precursor from benign tumors	Tavano et al. (2012)
Up	EGFR, HER2	Human, *In vitro*	PANC-1, MIA PaCa-2, BXCP-3	miR-21 associated with the EGFR) and human epidermal growth factor receptor (HER)2 pathways	Application of the miRNA panel investigated in the present study as a potential predictor of patient responses to anti-EGFR/HER2 treatment	Fang et al. (2022)
Up	-	Human tissue	-	Adjuvant gemcitabine after curative resection significantly and independently reduced disease-free survival in pancreatic cancer patients with LNA-ISH-detected miR-21 overexpression	miR-21 may serve as a significant predictor for gemcitabine resistance in patients	Dillhoff et al. (2008)

Abbreviations: ALDH1, Aldehyde Dehydrogenase1; BCL-2, B-Cell Lymphoma 2; BTG2, B-cell Translocation Gene 2; C5ORF41, Chromosome 5 Open Reading Frame 41; CD, cluster of differentiation; CDK, Cyclin-Dependent Kinase; Cstf3, Cleavage Stimulation Factor Subunit 3; CXCR4, C-X-C Chemokine Receptor Type 4; EFNA1, Ephrin-A1; EGFR, epidermal growth factor receptor; HPDE, non-tumorigenic human pancreatic ductal epithelial cell line; HER2, Human Epidermal Growth Factor Receptor 2; IRAK, Interleukin-1, Receptor-Associated Kinase; KLF, Kruppel-Like Factor; MAPK, Mitogen-Activated Protein Kinase; NRP, neuropilin; PDCD4, Programmed Cell Death 4; PI3K, Phosphoinositide 3-Kinase; PTEN, phosphatase and tensin homolog; Rasa, Ras P21 Protein Activator; RECK, reversion inducing cysteine rich protein with kazal motifs; SOCS, suppressor of cytokine signaling; Sox, SRY (Sex Determining Region Y)-Box; Spry2, Sprouty RTK, Signaling Antagonist 2; TPM, tropomyosin; Tgfbi, Transforming Growth Factor Beta Induced; Tgfbr, Transforming Growth Factor Beta Receptor; VHL, Von Hippel-Lindau; Wnt, Wingless-Type MMTV, integration site family member; ZBTB41, Zinc Finger and BTB, Domain Containing 41.

In pancreatic cancer, miR-21 plays a pivotal role in promoting tumor progression and chemoresistance, particularly through its effects on CSCs and EMT. Overexpression of miR-21 is closely associated with the downregulation of E-cadherin and the upregulation of mesenchymal markers such as N-cadherin, ZEB1, Snail, and vimentin, facilitating the EMT process. This leads to increased cell migration, invasion, and resistance to therapies like gemcitabine. Furthermore, miR-21 contributes to the modulation of key signaling pathways, including NF-B activation and non-canonical Wnt signaling, which in turn promotes the aggressive characteristics of PDAC. Despite the promising findings, there is a gap in understanding the exact molecular mechanisms through which miR-21 regulates EMT and CSCs in PDAC. Future studies should focus on validating these pathways in clinical settings, exploring miR-21 as a therapeutic target, and identifying biomarkers that can aid in the diagnosis and prognosis of PDAC.

### MircroRNA-21 and gastric cancer

**FIGURE 2:**
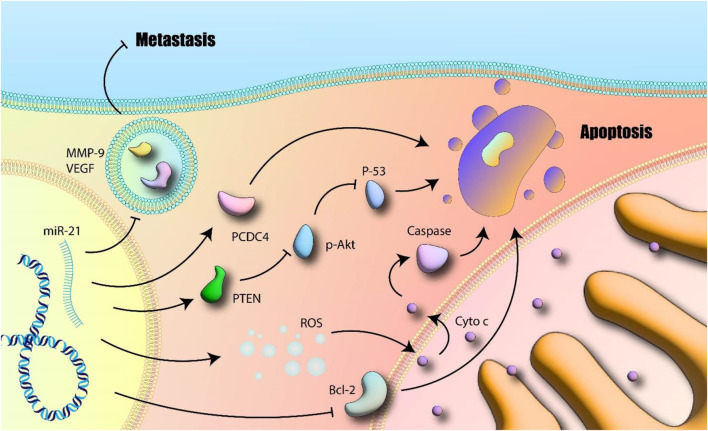
miR-21 and apoptosis: MiR-21 induces MMP-9 and VEGF and further promotes metastasis. Additionally, inhibition of apoptotic related mitochondrial pathway as well as promotion of subcellular signaling pathways which are involved in cell survival and proliferation are other suggested mechanisms. Akt-p: Phosphorylated Akt; BCL-2: B-cell lymphoma 2; Cytoc: Cytotoxic; MMP-9: Matrix Metalloproteinase 9; PTEN: Phosphatase and Tensin Homolog; ROS: Reactive Oxygen Species; VEGF: Vascular Endothelial Growth Factor (author-created).

A collection of genes essential for the onset and spread of cancer are the focus of Mir-21 (Paik et al., 2013). A key mechanism in gastric cancer involves miR-21 targeting PTEN (Wang et al., 2013) leading to activation of the PI3K/Akt pathway which correlates with infiltration, migration, and development (Khan et al., 2016). Qiang et al. look into whether curcumin and PD98059 work together to inhibit gastric cancer growth as a result of the miR-21/PTEN/PI3K/Akt pathway. At dosages that ranged from 5 to 40 M, it was discovered that curcumin had a time- and dose-dependent inhibitory impact on the proliferation of MGC-803 cells. Strong suppression of p-Akt protein expression was achieved with a high dosage of curcumin. PTEN expression went up and miR-21 went down when curcumin dosage was up. According to their findings, curcumin acted to suppress the miR-21/PTEN/Akt axis. Additionally, cell apoptosis produced by curcumin was substantially amplified after being pretreated with PD98059. The miR-21/PTEN/Akt pathway’s inhibition through curcumin has been also dramatically enhanced. Combining curcumin with PD98059 may be a successful stomach cancer treatment plan (Song WF. et al., 2013) (Figure 2).

#### Role in chemoresistance and autophagy via PI3K/Akt/mTOR

MiR-21 has recently been linked to Cisplatin (DDP)-resistant Gastric Cancer (GC) and has been found to be more abundant in these cells than in their parental comparable cells (Dong et al., 2011). Gu et al. investigated the link between miR-21, autophagy, and DDP resistance *in vitro*. They generated DDP-resistant human gastric adenocarcinoma cell lines by continuous DDP exposure. Western blot analysis confirmed activation of the PI3K/Akt/mTOR pathway in resistant cells. Autophagy levels were assessed by measuring Beclin-1 and LC3 expression (via Western blot) and quantifying autophagosome formation (e.g., via electron microscopy or fluorescent LC3 puncta). Cell survival and apoptosis were quantified using Annexin V-propidium iodide assays. GC cells developed resistance to the growth inhibition and death that the DDP therapy induced. Moreover, DDP-resistant GC cells had increased Akt and mTOR activity. When autophagy was suppressed, GC cells were less sensitive to DDP, but when autophagy was stimulated, the reverse outcome was shown. It was discovered that DDP-resistant GC cells had more microRNA miR-21 than the parent cells did. When DDP-resistant GC cells were transfected with a miR-21 inhibitor, the DDP-resistant GC cells became more sensitive through increasing autophagy, whereas when transfected with miR-21 mimics, DDP resistance has been restored by lowering autophagy. Both of these results were brought about by the miR-21 gene. They discovered that DDP resistance within GC cells is linked to the miR-21, which inhibits autophagy via the PI3K/Akt/mTOR pathway. This happens because miR-21 suppresses the process. Also, these findings suggested that the therapeutic targets for the successful treatment of DDP resistance within GC cells may include the autophagy-causing substances (Zhao et al., 2015). This study directly links miR-21 overexpression to chemoresistance (specifically to cisplatin) by demonstrating its ability to suppress protective autophagy via the PI3K/Akt/mTOR pathway, identifying autophagy induction as a potential strategy to overcome miR-21-mediated resistance. Inflammatory signaling pathways are also key regulators and effectors of miR-21 in gastric cancer.

#### Regulation by inflammatory signaling (NF-κB, IL-6, STAT3)

MiR-21 expression is controlled more than usual by NF-kappaB activation within bone marrow-derived macrophages (Hwang et al., 2010), also through myeloma cells especially inflammatory cytokines interleukin (IL)-6 (Toste et al., 2015). Through the related activator of transcription 3 (Stat3) and inactive signal transducer as well as the common gp130 co-receptor subunit, miR-21 expression and IL-6 signaling were related (Zhang et al., 2019a). Stat3 has a substantial impact on the development, stability, and advancement of solid tumors. This is partially because it is a negative regulator of the immune system’s response to malignancies and an intrinsic promoter of neoplastic cells (Yuan et al., 2016). Patients with gastric cancer generally have a worse prognosis when their Stat3 levels are high, and this is especially true if the disease has spread to their lymph nodes (Mace et al., 2013). Tse et al. demonstrated that miR-21 is a Stat3-regulated driver in tumor growth along with enhancement using the development of inflammatory mediator’s gastric cancers in Gp130 F/F mice. This technique was used to demonstrate that Stat3 was necessary for the development of the tumor. Their results were managed to bring into agreement when they identified multiple preserved Stat3 binding sites upstream of the miR-21 gene promoter and demonstrated that systemic administration of a miR-21-specific antisense oligonucleotide antagomir lowered established gastric tumor volume in the Gp130 F/F mouse model of inflammation-associated gastric cancer. Inhibition of miR-21 reduced markers of EMT, decreased ECM remodeling, and restored PTEN function both in cell culture (*in vitro*) and in the mouse model (*in vivo*). All of these advantages result from reactivating PTEN. Gastric cancer patients with high levels of STAT3 as well as miR-21 have a worse prognosis, according to preclinical research. A powerful therapeutic target for solid tumors with increased Stat3 activity, miR-21 encourages the development of gastric cancer related to inflammation. For solid tumors with strong Stat3 activation, miR-21 is a potential therapeutic target (Rachagani et al., 2015). The identification of miR-21 as a direct transcriptional target of STAT3 firmly places it within the inflammation-cancer axis, suggesting that the pro-tumorigenic effects of chronic inflammation (e.g., mediated by IL-6) in the stomach may be, at least in part, channeled through miR-21 upregulation. Clinical studies have further explored the potential of miR-21 isoforms as diagnostic and prognostic markers.

#### Clinical significance of miR-21-3p as a biomarker

Inflammatory illnesses have been shown to have a connection to miR-21-3p, according to earlier research. miR-21-3p is the primary driver of colorectal cancer because it controls the signaling pathways involved in inflammation (Vila-Casadesús et al., 2018). Also, while renal fibrosis is developing, a higher amount of miR-21-3p in the body produces an increase in the inflammatory response (Benesova et al., 2020). According to in human study of Calsina et al., miR-21-3p may influence a patient’s prognosis if they have metastatic paraganglioma or pheochromocytoma (Ryu et al., 2011). In another in human study, Sun et al. investigated the role of miR-21-3p in the occurrence of gastric cancer and the prognosis of the patient (GC). Analyzing One hundred GC patients. miR-21-3p was tested in primary GC and paracancer mucosa. Receiver Operating Characteristic (ROC) curves for miR-21-predictive 3p in GC. Tumor size, lymph node metastases, and TNM staging subdivided GC patients. GCs produce the miR-21-3p. Stage, size, and lymph node metastases were divided into categories. Patients with big tumors, lymph node metastases, or in the GC tissues of patients with advanced TNM staging, miR-21-3p levels were greater. MiR-21-3p can diagnose GC, per ROC curves. In GC patients overexpressing miR-21-3p, Progression-Free Survival (PFS) and Overall Survival (OS) were worse. Size, lymph node metastases, TNM staging, and miR-21-3p level impacted GC prognosis (LaConti et al., 2011). Consistent clinical findings correlating elevated miR-21 levels (including specific isoforms like miR-21-3p) with advanced stage and poor prognosis reinforce its potential utility as a non-invasive biomarker for gastric cancer diagnosis and patient stratification. Mechanistically, miR-21 also impacts cancer metabolism.

#### Impact on metabolism via PDHA1 regulation

The enzyme pyruvate dehydrogenase alpha 1(PHA1) is essential. This enzyme connects glycolysis with the citric acid cycle in the mitochondria by catalyzing pyruvate decarboxylation. Cancer cells become more lethal when pyruvate dehydrogenase is inhibited, since this enhances the Warburg effect (Papaconstantinou et al., 2013; Nagao et al., 2012). Cancer cells have been shown to undergo metabolic reprogramming when there is deregulation of the gene PDHA1, which has also been discovered (Liu P. et al., 2013). Poor ovarian cancer prognosis has been connected to low PDHA1 expression, according to the literature (Wang F. et al., 2015). Moreover, the more aggressive features of clear cell carcinoma have been associated to low amounts of PDHA1 protein expression (Popov et al., 2015). This work reveals a metabolic dimension to miR-21’s oncogenic function, linking its overexpression to the Warburg effect via suppression of *PDHA1* and suggesting that targeting miR-21 might impact tumor metabolism. Beyond metabolism, miR-21 continues to be implicated in EMT regulation within gastric cancer.

#### Promotion of EMT via TGF-β1/PTEN axis

Li and colleagues found *in vitro* that miR-21 promotes TGF-β1-induced EMT in gastric cancer cells, an effect linked to regulation of PTEN expression. Inhibition of miR-21 using specific inhibitors led to increased expression of PTEN and E-cadherin, while decreasing the levels of N-cadherin, β-catenin, Vimentin, and Slug, markers associated with EMT. The inhibition of miR-21 significantly reduced cell migration and invasion, both *in vitro* (through scratch tests) and *in vivo* (xenograft models), by regulating EMT-related factors. These findings suggest that miR-21 facilitates TGF-β1-induced EMT in GC cells via its known targeting of PTEN, thus promoting tumor progression (Park et al., 2009). Liu et al. looked into gastric cancer by studying the miRNA that controls PDHA1. Researchers found a connection between PDHA1 downregulation and a bad prognosis for gastric cancer. Decreased levels of PDHA1 increased glycolysis, which promoted gastric cancer growth and metastasis. To boost glycolysis and cell growth in gastric cancer, miR-21-5p selectively addressed PDHA1 and downregulated PDHA1 expression. MiR-21-5p was elevated in specimens of gastric cancer, while PDHA1 expression was inversely linked with it. Finally, they suggested that miR-21-5p may target PDHA1 to control a metabolic shift and cancer progression in gastric cancer, suggesting a possible function for this pathway in the therapy of gastric cancer (Hong and Park, 2014). Collectively, the studies on gastric cancer consistently implicate miR-21 in promoting invasion, metastasis, chemoresistance, and metabolic reprogramming, frequently converging on the PTEN/PI3K/Akt pathway, while also highlighting its connection to inflammatory signaling and its potential as a clinical biomarker. This pathway, essential for cell proliferation and survival, also regulates autophagy, which plays a paradoxical role in cancer progression and therapy response. Targeting the miR-21/PTEN/Akt signaling axis offers a potential therapeutic approach for GC, and further studies are needed to optimize such strategies for clinical application. Table 2 lists various studies on miR-21 and gastric cancer.

**TABLE 2:** miR-21 and gastric cancer.

Type of miR-21	Status of expression	Target	Model	Cell line type	Effect	Conclusion	Ref.
miR-21	Up	KLF5, RECK, TMP1, PDCD4, PTEN, BAX, BCL-2	*In vitro*, *In vivo*	AGS, MKN1	Inhibition of miR-21 has therapeutic benefits with the functional restoration of PTEN *in vitro* and *in vivo*	miR-21 as a robust therapeutic target for solid malignancies characterized by excessive Stat3 activity	Rachagani et al. (2015)
miR-21-3p	Up	-	Human, *in vitro*	SNU-1, AGS, Hs 738.St/Int	He expression levels of miR-21-3p were markedly increased in cancer tissues	Overexpression of miR-21-3p promoted cancer cell migration and invasion	Wang et al. (2009a)
	Up	-	Human	-	miR-21 and miR-25 were significantly upregulated in GC patients	miR-21 in plasma samples can be served as a potential noninvasive tool in detection of GC	Joshi et al. (2014)
	Up	-	*In vitro*, *In vivo*	SGC-7901	The GCIF was able to upregulate the expression of miR-21 in the subcutaneously transplanted tumors		Wartenberg et al. (2016)
	Up	WWP1, SKP2, KLHL42, FBXO11	Human	-	miR-21 was significantly higher in gastritis group	Overexpression of miR-21may indicate a connection between miRNAs and H. pylori-related problems	Ali et al. (2010)
	Up	-	Human	-	Significant upregulation of miR-21 in the EGN mucosa	oncogenic potential for miR-21	Ryu et al. (2010)
miR-21-5p	Up	LIFR	*In vitro*, *In vivo*	GES-1, AGS, HGC-27	miR-21-5p overexpression attenuated Lidocaine-induced anti-proliferative and anti-metastatic effects on GC cells	Lidocaine might GC cell malignancy by modulating circ_ANO5/miR-21-5p/LIFR axis, highlighting a novel insight for GC treatment	Valladares-Ayerbes et al. (2011)
miR-21-5p	Up	RUNX1	Human, *In vitro*, *In vivo*	AGS, HGC-27, GES-1	circ_0027599 positively regulated RUNX1 expression via functioning as the sponge for miR-21-5p	Circ_0027599 modulates the miR-21-5p/RUNX1 axis, which may reveal a new GC treatment target.	Carter et al. (2016)
miR-21	Up	APE1, ATM, ATR	*In vitro*	AGS, HGC-27	Upregulation miR-21	It may be a promising strategy for controlling tumor progression	Ali et al. (2015)
miR-21-5p	Up	-	Human	-	Higher miR-21-5p expression was associated with T3 + T4 and stage III + IV.	miRNAs may act as potential diagnostic markers	Karamitopoulou et al. (2017)
	Up	-	Human, *In vitro*	SNU-398, SNU-182		Upregulating lncRNA CASC11 upregulates miR-21 to cause carboplatin resistance in HCC patients	Habbe et al. (2009)
	Up	PTEN, ERK	Human, *In vitro*	MKN28, MKN45	miR-21 expression was upregulated in GC tissues and could be negatively regulated by circHIAT1	CircHIAT1 downregulated miR-21 to suppress GC cell tumors, suggesting it may be a potential GC therapy target.	Calatayud et al. (2017)
	Up	PI3K	*In vitro*	AGS/DDP	miR-21 mimics contributed to restored DDP resistance by suppressing autophagy	miR-21 is associated with DDP resistance in GC cells by inhibiting autophagy via the PI3K/Akt/mTOR pathway	Zhao et al. (2015)
miR-21-5p	Up	-	Human, *In vitro*, *In vivo*	MKN45, SGC-7901, BGC-823, GES-1	miR-21 is a GAS5 target and GAS5 inhibits the proliferation of gastric cancer cells by targeting miR-21	Targeting miR-21 expression may effect on migration, invasion, and tumor formation, and increase apoptosis	Bhatti et al. (2011)
miR-21	Up	-	*In vitro*	MGC-803, MKN-28, AGS, BGC-823, GES-1	miR-21 expression in GC cell lines was higher than in a gastric mucosal epithelial cell line	miR-21 might promote the invasion and metastasis of GC by upregulating EMT.	Paik et al. (2013)
miR-21	Up	KCNK15-AS1	Human, *In vitro*	GES-1, BGC-823, SGC-7901	Knockdown of the expression of miR-21 inhibited proliferation and promoted apoptosis	KCNK15-AS1 interacted with miR-21	Duell et al. (2017)
	Up	-	*In vitro*	MKN-45		miR-21, which are involved in stemness and chemo-radioresistance, may provide novel GC therapy insights	Humeau et al. (2015)
	Up	PTEN, NF‐κB	*In vitro*	MKN-45	(miR-21) increased cell viability, migration, and invasion	Celastrol reduces MKN45 cell proliferation, migration, and invasion via down-regulating miR-21 and inactivating PTEN/PI3K/AKT and nuclear factor κB signaling pathways	Liu et al. (2012)
	Up	SATB1, TIAM1, PDCD4, PTEN, APAF1, TIMP3, TGF-β, PLAG1	Human	-	miR-21 and miR-222 were significantly higher in GC plasma	miR-21 and miR-222 in plasma samples can be served as a potential noninvasive tool in GC detection	Xue et al. (2013)
	Up	PTEN	*In vitro*	MGC-803	Colonospheres that are highly enriched in cancer stem/stem like cells reveal increased miR-21 expression and decreased PTEN	CDF normalizes miR-21-PTEN-Akt pathway suggests that the chemical may treat chemotherapy-resistant colorectal cancer	Song et al. (2013a)
	Up	PTEN	*In vitro*	AGS, NCI-N87	miR-21 inhibited cell cycle progression and enabled apoptosis	miR-21 may provide a therapeutic target for treatment of human gastric cancer	Tian et al. (2016)
	Up	15-PGDH	Human, *In vitro*	TMK-1, AGS, KATO III, NCI-N87, MKN-1, MKN-28, MKN-45, SNU-1, SNU-5, SNU-216, SNU-484, SNU-601, SNU-638, SNU-668, SNU-719	miR-21, which was detected in high level in gastric tumors	Loss of 15-PGDH occurs at the very early stage of gastric adenocarcinoma by miR-21. *H. pylori* infection may affect miR-21 upregulation	Morinaga et al. (2016)
	Up	DAXX	Human, *In vitro*	NCI-N87, KATOIII, AGS, MKN28	These sponges inhibit cancer cell proliferation and suppress the activity of miR-21 on downstream protein targets	miR-21could be used as potential future therapeutic application in human patients	Zhang et al. (2012)
miR-21-5p	Up	PDHA1	Human, *In vitro*	GES-1	miR-21-5p was significantly upregulated in gastric cancer	Potential role of the miR-21-5p/PDHA1 axis in gastric cancer treatment	Hong and Park (2014)
	Up	-	Human	-	The level of miR-21 showed no significant differences among patients with different clinical and pathological characteristics	The identification of serum miR-378 and miR-21 affects GC diagnosis	Wang et al. (2014)
	Up	-	Human	-	miR-21 were significantly related to an advanced TNM stage in GC patients	Circulating miR-21, could be used as diagnostic plasma biomarkers in gastric cancer patients	Roy et al. (2013)
	Up	-	Human	-	Increased expression levels of miR-21 in GC samples	Some miR-21 changed in GC may be researched as biomarkers for early diagnosis and prognosis	Qiang et al. (2019)
	Up	-	Human	-	miR-21 and miR-145 was significantly associated with worse prognosis of gastric cancer patients	miR-21 has the potential to serve as relevant diagnostic and prognostic biomarkers of GC	Shen et al. (2014)
	Up	PTEN	Human	-	miR-21 were upregulated in GC patients with *H. pylori*infection	Suggests that the miRNAs and genes may help diagnose GC.	Liu et al. (2016c)
	Up	MMP-3, MMP-9, VEGF	*In vitro*, *In vivo*	AGS, NCI-N87, SGC-7901, MKN-45, TMK-1, GES-1	Overexpression of miR-21 promoted cell mobility of AGS through activation of EMT	MEG3/miR-21 axis participates in the tumor progression and metastasis of gastric cancer through the regulation of EMT.	He et al. (2015)
	Up	-	Human, *In vitro*, *In vivo*	MKN74, MKN45, SGC7901, AGS, GES-1	pcDNA3.1-MEG3 transfection could counteract the promoting role of miR-21 mimic on GC cell proliferation and metastasis	Regulating role of MEG3/miR-21 axis in GC progression and provided a new potential therapeutic strategy for GC treatment	Yang et al. (2013a)
miR-21-5p	Up	caspase-8, PI3K, PTEN	*In vitro*, *In vivo*	BGC823, SGC7901	miR-21 regulated by NF-κB mediated the expression of P-gp protein via inhibiting caspase-8, thus regulating Cisplatin-induced cell death	Our results suggest that LV-METase has potential as a therapeutic agent for gastric cancer treatment	Li et al. (2016b)
	Up	PTEN, P53	Human, *In vitro*, *In vivo*	MKN28, MKN45, SGC-7901, AGS, NCI-N87, HGC-27	CBX7 was found to upregulate miR-21 via the activation of AKT-NF-κB pathway	CBX7 positively regulates stem cell-like characteristics of gastric cancer cells by inhibiting p16 and activating AKT-NF-κB-miR-21 pathway	Vahidnezhad et al. (2016)
	Up	p53, PDCD4, Bcl-2	Human, *In vitro*	MGC803	Inhibition of miR-21 reduced the levels of *miR-21* and *Bcl-2* in MGC803 cells	miR-21 and Bcl-2 may be biomarkers and therapeutic targets for gastric cancer	Gu et al. (2020)
miR-21-5p	Up	PTEN, TIMP3	Human, *In vitro*	SGC7901, HEK-293T	Suppressing miR-21-5p expression partially sensitized SGC7901/DOX cells to DOX	Suggests the potential utility of miR-21-5p antagonism to sensitize GC cells to DOX chemotherapy	Sheedy et al. (2010)
	Up	PTEN	Human	-	-	miR-21 can be use as potential biomarker for GC.	Löffler et al. (2007)
	Up	PTEN	*In vitro*	MKN74	miR-21 inhibition was associated with increased expression of PTEN	miR-21 inhibitor may provide a novel therapeutic strategy for gastric cancer	Iliopoulos et al. (2010)
	Up	15-PGDH	Human, *In vitro*	BGC-823, MKN-28, AGS	miR-21 is negatively correlated with 15-PGDH	miR-21 play a role in the progression of gastric cancer	Johnson et al. (2018)
miR-21-5p	Up	-	Human	-	miR-21-5p could be detected in small amounts of urine sample	Urine miR-21-5p could be utilized as a novel non-invasive biomarker of gastric cancer detection and monitoring	Deng et al. (2010)
	Up	-	Human, *In vitro*	MGC-803	miR-21 and miR-182 in peripheral blood of gastric cancer patients were significantly high	Serve as a target for the clinical treatment of gastric cancer	Tse et al. (2022)
	Up	-	Human	-	miR-21 was significantly higher in the peripheral circulation	Serum miRNA biomarkers may originate from tissues other than the primary tumour	Li et al. (2016b)
	Up		Human	-	miR-21 expression in tissue was associated with tumor differentiation	miR-21 could serve as a potential biomarker to identify MGC with chemoresistance	Butz et al. (2016)
	Up	p53, PTEN	Human, *In vitro*	SGC7901, MKN45, GES-1, AGS	There was a positive correlation between Bmi-1 and miR-21 expression in gastric cancer	Bmi-1 upregulates miR-21 and miR-34a by activating AKT-NF-kB pathway	Calsina et al. (2019)
	Up	PTEN	Human, *In vitro*, *In vivo*	SGC-7901, KATO-III	MiR-21 inhibitors significantly inhibit cell migration and invasion in GC cell lines	miR-21 could promote TGF-β1-induced EMT in GC cells through up-regulating PTEN expression	Park et al. (2009)
	Up	-	Human	-		miRNAs is a novel and noninvasive biomarker for gastric cancer, and could facilitate and simplify its diagnosis	Sun et al. (2020)
	Up	-	Human	-		miRNAs as promising biomarkers for detection of patients at early stages	Kim et al. (2006)
	Up	FZD6	*In vitro*	SGC-7901, AGS, GES-1	miR-21- effects in the canonical and non-canonical wnt pathways	May be an important implication for future therapy	Dupuy et al. (2015)
	Up	-	Human	-	Pregulated in malignant versus adjacent benign gastric mucosa	Suggest the potential for a noninvasive addition to cancer diagnostics	Li et al. (2016c)
	Up	Noxa	Human, *In vitro*	SGC-7901	An increased miR-21 expression level was identified as a risk factor for advanced stage gastric cancer	miR-21 expression may induce gastric cancer migration and invasion via the downregulation of Noxa expression	Liu et al. (2015)
miR-21-5p	Up	-	Human	-		miR-21-5p may be useful as a predictor of recurrence in young GC patients whose tumors contain a high proportion of intratumoral stroma	Li et al. (2016d)
	Up	-	Human	-			Lin et al. (2016)
	Up	Bax, Bak, PTEN, Bcl-2	*In vitro*	AGS	miR-21 mediates anticancer effects of NS398 in GC cells by regulating apoptosis-related proteins	miR-21 is one of the molecular targets of this specific cyclooxygenase-2 inhibitor in the prevention and treatment of GC	Liu et al. (2018b)
	Up	-	Human	-	Higher tissue hsa-miR-21 level was related to a lower overall survival rate	Can be potentially applied as novel and non-invasive biomarkers for GC.	Li et al. (2022)
	Up	-	Human	-		Can be used for early detection of gastric cancer	Cuellar-Gomez et al. (2021)
	Up	PTEN	Human	-	miR-21 in both serum and PBMCs increased significantly in GC patients	miR-21 (both in serum and in PBMCs) can serve as a good biomarker for GC and could be used in diagnosis of early (stage I) and late GC (stage IV)	Sun et al. (2021)
	Up	-	Human	-	Stromal miR-21 is closely related to tumour progression in GC	Stromal miR-21 of tumours might be a target of treatment	Nooh et al. (2021)
	Up	PTEN, PDCD4	*In vitro*	SGC-7901, MKN-45	miR-21 inhibitor increased the expression of PTEN and PDCD4 proteins and significantly reduced cell proliferation, migration and invasion	May have important role in gastric cancer growth and dissemination by modulating the expression of the tumor suppressors PTEN and PDCD4	Pita et al. (2021)
	Up	-	Human	-	was significantly overexpressed in gastric tumors compared to normal gastric tissues	Potential as prognostic biomarkers in late stage gastric cancer	Guan et al. (2022)
	Up	-	Human	-	miR-21 level was associated with the tumor node metastasis (TNM) stage, tumor size and tissue categories	miR-21 in peripheral blood as a novel tool for monitoring CTCs in gastric cancer patients	Han et al. (2021a)
	Up	-	Human	-	miR-21 in gastric cancer was significantly high	Potential as prognostic biomarkers in gastric cancer	Manoel-Caetano et al. (2020)
	Up	PDCD4	Human, *In vitro*	MKN1, MKN7, MKN45, MKN74, NUGC3, NUGC4 AZ521, KATOIII	An inverse correlation between PDCD4 mRNA and miR-21 was found in gastric cancer	May serve as a target for effective therapies	Zhang et al. (2020)
	Up	RECK	Human, *In vitro*	AGS SGC7901, MKN45, MKN28 GES-1, HEK-293T	Forced expression of miR-21 significantly enhanced cell proliferation and invasion	miR-21 may be important in the initiation and progression of gastric cancers as an oncomiR	Liu et al. (2020b)
	Up	Serpini1	Human, *In vitro*	MKN28	miR-21 and Serpini1 expression levels were inversely correlated in a subgroup of gastric cancers	miR-21 is upregulated, inducing downregulation of Serpini1	Quan et al. (2020)
	Up	PDCD4, RECK, PTEN	Human	-			Li et al. (2020)
	Up	PTEN	*In vitro*	MKN-45	Increase in mir-21 and mir-302 expression level in CSCs, relative to cancer cells	May be promising objects for targeting CSCs specifically and efficiently	Xiao and Jie (2019)
	Up	PTEN	Human, *In vitro*	SGC-7901, MKN-28, MKN-45, AGS	The transwell test indicated that cell migration *in vitro* was notably inhibited with the downregulation of miR-21	miR-21 suppression may increase PTEN expression, suggesting that gastric cancer may start and progress via PTEN.	Wang et al. (2013)
	Up	PDCD4	Human, *In vitro*	AGS, MKN1, SNU216, SNU484, SNU638	miR-21 overexpression was frequently detected in gastric cancers	May play a role in the development and progression of gastric cancers	Zhang et al. (2019b)
	Up	FASLG, BTG2	*In vitro*, *In vivo*	GES-1, AGS, BGC-823, HGC-27, MKN-28, SGC-7901	miR-21, contribute to the transformation induced by MNNG in GES-1 cells	Offers a new explanation of the mechanisms underlying chemical carcinogenesis	Motamedi et al. (2020)
	Up	-	Human	-	Expression of miR-21in gastric cancer samples was significantly high	May be applicable to future decisions regarding treatment or as a diagnostic biomarker	Yao et al. (2019a)
	Up	-	Human	-	The plasma miR-21 expression was highly associated with differentiation degree and lymph node metastasis rate	miR-21 could be a novel potential biomarker for GC prognosis	Emami et al. (2019)
	Up	PI3K	*In vitro*	GES-1, MGC-803, BGC-823	miR-21 inhibitor could decrease phospho-Akt expression and NF-κB activity	The effect of celastrol on apoptosis was due to miR-21 inhibiting the PI3K/Akt-dependent NF-κB pathway	Zhou et al. (2019)
	Up	-	Human	-	miR-21 in the serum were associated with an increased tumor size and an advanced pT stage	Serum miR-21 could be exploited as a practical biomarker for monitoring tumor burden in patients with GC.	Park et al. (2018)
	Up	PTEN	*In vitro*	MKN45, NUGC4, NCI-N87	miR-21/PTEN pathway regulated the sensitivity of HER2-positive GC cell lines to trastuzumab through modulation apoptosis	Suggest miR-21/PTEN pathway may be essential to the trastuzumab resistance mechanism in GC.	Liu et al. (2018c)
	Up	-	Human	-	miR-21 after eradication were significantly higher in the high-risk group than in the controls	May provide a novel and stable marker of increased risk for early gastric cancer after *H. pylori* eradication	Huang et al. (2018a)
	Up	-	Human	-	miR-21 was detected in patients with low social status	May show homogenous correlations with the existence of common risk factors	Zhao et al. (2018c)
	Up	PDCD4	Human	-	The relative expression of PDCD4 was negatively correlated with miR-21	ROS increases gastric carcinogenesis by upregulating miR-21, which downregulates PDCD4 in gastric cancer cells	P et al. (2018)
	Up	PTEN, PDCD4, RECK	*In vitro*, *In vivo*	U87, LN229, MCF-7, MDA-MB-231, SGC7901	Particular small-molecule miR-21 inhibitor, AC1MMYR2, which prevented Dicer from processing pre-miR-21 to mature miR-21	Could be used as a broadly useful candidate antitumor drug	Obermannova et al. (2018)
	Up	PTEN, RECK, PDCD4	Human, *In vitro*	AGS, HGC-27, GES-1	Caudatin downregulated the expression of oncomir miR-372 and miR-21	Wnt/β-catenin signaling is a novel mechanism of action for caudatin during therapeutic intervention in gastric cancers	Ranjbar et al. (2018)
	Up	PTEN	*In vitro*	SGC7901			Dong et al. (2011)
	Up	-	Human	-	Deregulation of miR-21 (upregulation) was detectable in both gastric and esophageal adenocarcinomas	Candidates that can be investigated for their biological functions and for their possible diagnostic	Xu et al. (2018)
	Up	-	Human	-	miR-21 levels in intestinal type gastric cancer specimens were higher than that in diffuse	miRNAs in gastric juice are potential biomarkers that can assist in screening for gastric cancer	Dan et al. (2018)
	Up	-	Human	-	miR-21 were significantly higher in GC patients with stage I	Novel potential biomarkers for GC detection	Xin et al. (2018)
	Up	PDCD4, Serpin B5	Human	-	-	-	
	Up	-	Human	-	miR-21 level and multiple clinicopathological factors	Elevated miR-21 is linked to lymph node metastases, which could be used as a biomarker in GC patients	Gu et al. (2018)
	Up	TPM1, PDCD4, PTEN, RECK	Human	-	miR-21 was significantly overexpressed in human solid cancerous serum	MiR-21 may be a broad-spectrum serum biomarker for various solid tumors.	Chen et al. (2018a)

Abbreviations: 15-PGDH, Prostaglandin Dehydrogenase 15; APAF1, Apoptotic Protease Activating Factor 1; APE1, Apurinic/Apyrimidinic Endonuclease 1; ATM, ataxia telangiectasia mutated; ATR, Ataxia Telangiectasia and Rad3-Related; Bak, Bcl-2, antagonist killer; BAX, Bcl-2-Associated X Protein; BCL-2, B-Cell Lymphoma 2; BTG-2, B-cell Translocation Gene 2; DAXX, Death Domain-Associated Protein; ERK, Extracellular Signal-Regulated Kinase; FASLG, fas ligand; FBXO11, F-box Protein 11; FZD6, Frizzled Class Receptor 6; HPDE, non-tumorigenic human pancreatic ductal epithelial cell line; KCNK15-AS1, Potassium Two-Pore Domain Channel Subfamily K Member 15 Antisense RNA, 1; KLF, Kruppel-Like Factor; KLHL42, Kelch Like Family Member 42; LIFR, leukemia inhibitory factor receptor; MAPK, Mitogen-Activated Protein Kinase; MMP, matrix metalloproteinase; NF-κB, Nuclear Factor Kappa B; PDCD4, Programmed Cell Death 4; PI3K, Phosphoinositide 3-Kinase; PLAG1, PLAG, Transcription Factor 1; PTEN, phosphatase and tensin homolog; RECK, reversion inducing cysteine rich protein with kazal motifs; RUNX1, Runt-Related Transcription Factor 1; SATB1, Special AT-rich Sequence-Binding Protein 1; SKP2, S-phase Kinase-Associated Protein 2; TGF-β, transforming growth factor beta; TIAM1, T-Cell Invasive Antigen 1; TIMP3, Tissue Inhibitor of Metalloproteinases 3; TPM, tropomyosin; VEGF, vascular endothelial growth factor; VHL, Von Hippel-Lindau; Wnt, Wingless-Type MMTV, integration site family member; WWP1, WW, Domain Containing E3 Ubiquitin Protein Ligase 1.

### MircroRNA-21 and esophageal cancer

The cell surface is covered with a protein family called cell adhesion molecules (CADMs). Members of the CADMs family include CADM1, CADM2, CADM3, and CADM4. The CADMs family of genes that inhibit tumors has been discovered (Huang Y. et al., 2018). Some forms of malignant tissue either completely lack or express CADMs at very low levels. For instance, in lung cancer and prostate cancer, promoter methylation prevents the formation of CADM1 (also known as TSLC1) (Wang P. et al., 2018). Also, in prostate cancer, CADM2 expression is downregulated (Li L. et al., 2018). The authors (Li et al.) examine the underlying mechanism of action of CADM2 and its possible significance in ESCC. In this work, In ESCC cell lines and tissues, the scientists discovered that CADM2 expression was comparatively low. The overexpression of CADM2 prevented the growth of ESCC cells and caused them to commit suicide. Additionally, overexpression of CADM2 prevented ESCC cells from using the Akt signaling pathway. Anti-miR-21-5p was observed to limit cell proliferation as well as trigger apoptosis after miR-21-5p has been downregulated, whilst CADM2 knockdown attenuated these effects. Anti-miR-21 transfected cells had decreased levels of p-Akt expression. Si-CADM2 co-transfection increased the expression of p-Akt in the cells within comparison to cells transfected via anti-miR-21-5p. Downregulation of miR-21-5p prevents ESCC cell proliferation as well as death via the CADM2/Akt pathway. According to their research, treating ESCC by focusing on the miR-21-5p/CADM2-Akt axis may offer a fresh, successful method (Kao et al., 2017). These findings identify the miR-21-5p/*CADM2*/Akt axis as a specific regulatory node controlling ESCC proliferation and apoptosis, offering a potential therapeutic target.

#### Interaction with the immune microenvironment and T cells

The presence of specific T cell subsets as well as the organized growth and activation of T lymphocytes in the tumor microenvironment are crucial for cancer immunosurveillance (Wang et al., 2017). Cytotoxic CD8^+^ T cells, which form the majority of tumor-infiltrating lymphocytes, are key effectors of anti-tumor immunity, which may be further subdivided into the following groups: CD8^+^ Tregs, Tc1, Tc2, Tc17, and Tc18 (Sierzega et al., 2017). Interferon gamma (IFN) as well as tumour necrosis factor alpha (TNF) are created by Tc1 cells and have a strong anti-tumor impact, but interleukin (IL)-4, and IL-10, as well as IL-5, secreted through Tc2 cells have a minor to no effect on tumor formation (Qi et al., 2017; Wang et al., 2016b). Because Tc17 CD8^+^ T cells do not exhibit cytotoxic effects, it is unknown if functional and phenotypic factors contribute to their antitumor activity. In-depth research on Tc17 CD8^+^ T cells (IL17 secreting) is lacking (Huang et al., 2016; Mirzaei et al., 2016). An increase in CD8^+^ Treg cells was connected to downregulated anti-tumor immune reactions in some tumour microenvironments (Yan et al., 2016). T-cell regulation is one of the immune cell development and activation mechanisms that microRNAs control (Treece et al., 2016; Sun et al., 2016). The tumor suppressor gene PTEN is primarily targeted by the oncogene miR-21, which encourages the invasion and growth of ESCC cancer cells (Park et al., 2016). This gene was identified as a possible therapeutic target and a promising candidate for the creation of reliable prognostic and diagnostic biomarkers in ESCC due to its high expression (Sisic et al., 2015). MiR-29b, on the other hand, is hypothesized to function as a tumor-suppressor miRNA because it is typically downregulated in ESCC (Li H. et al., 2015). In another study conducted by Samiei et al.; they compared CD3^+^CD8^+^ T cells from 34 patients who have ESCC (12 recently diagnosed: ND, 24 under-treated: UT) to CD3^+^CD8^+^ T cells from 34 matched healthy donors to ascertain the diagnostic and/or prognostic utilities of IL-10, IFN-γ, and TGF-β, as well as IL-17a producing CD8^+^ CD3^+^ T cells (after 160 weeks of follow-up) (Wang D. et al., 2015). Only patients with UT had an increase in Tc17 and CD8^+^ Treg cell frequency, whereas ND and UT ESCC patients also experienced an increase within IL-10 and TGF-producing CTLs (CD8^+^ Tregs). Area under the curve [AUC] >0.9 analysis revealed TGF and IL10 expression medians in CTLs to be highly distinguishing biomarkers among ESCC patients and healthy controls. Further, decreased expressions of TGF, IL17a, IL10, and IFN in CTLs were linked to improved prognosis in ESCC. Novel treatment goals in addition potent Predictive and Diagnostic tools for ESCC may be found in the correlation between miR-21 expression and the decreased function of CD3^+^ CD8^+^ T cell subsets (Wang D. et al., 2015). This study uniquely links elevated miR-21 expression in ESCC patients to dysregulation of anti-tumor cytotoxic T cell function, suggesting miR-21 contributes to immune evasion in the tumor microenvironment. Returning to intrinsic cellular pathways, the PTEN/PI3K/Akt axis is a frequent target.

#### Regulation of the PTEN/PI3K/Akt pathway

It is now understood that this signal pathway is aberrant in conditions such thyroid cancer, triple-negative breast cancer, cerebral cavernous malformations, and prostate cancer (Wu et al., 2015). WU et al. investigated how miR-21 regulation influences the biological functions of human esophageal cancer cells. They found miR-21, PI3K, and Akt expression were higher, while PTEN expression was lower, in esophageal cancer tissues compared to adjacent normal tissues. A low positivity for PTEN protein was found in lymph node-metastatic, poorly differentiated esophageal cancer tissues, but positivity for PI3K and AKT proteins was widespread. When compared to the negative and blank groups, we find that inhibition-miR-21 group considerably upregulates the PTEN expression in TE11 cells. Moreover, the Inhibition-miR-21 group drastically decreased TE11 cell invasion, migration, and proliferation in comparison to the empty and negative control groups. The amount of TE11 cells in the cell cycle’s G0/G1 stage was considerably more in the inhibition-miR-21 group comparing to the control group, although the cells’ proportion in S and G2/M phases was considerably lower. TE11 cells have much greater apoptosis rates than the other cell lines examined. The study concluded that miR-21 promotes esophageal cancer cell growth, migration, invasion, and survival while suppressing apoptosis, effects mediated via the PTEN/PI3K/Akt pathway (Jiang et al., 2011). The role of miR-21 in treatment response is also under investigation. Reinforcing findings from other GI cancers, this work confirms the central role of the miR-21/PTEN/PI3K/Akt pathway in promoting esophageal cancer cell proliferation, migration, and invasion, while inhibiting apoptosis.

#### Involvement in radiosensitivity and target gene regulation (PDCD4, KRAS)

The rising amount of research demonstrating that miRNA expression and the prognosis of esophageal cancer are tightly related and it has revealed that miR-21 may promote radiosensitivity in esophageal carcinoma down TE-1 cells (Zheng et al., 2011). The objective of the research conducted by Wen et al. was to examine the miR-21 expression along with the impacts that it has upon cancer cell invasion, apoptosis, and the target genes’ expression in order to better understand its function in esophageal cancer. In order to determine the production of miR-21 in human esophageal tissues, surrounding tissues, as well as an esophageal cancer cell line, a fluorescent quantitative polymerase chain reaction experiment has been conducted. Protein expression levels of the miR-21 targets PDCD4 and PTEN, as well as KRAS, were examined by Western blotting. These findings revealed a large rise in mir-21 expression within TE-13 cells as well as esophageal cancer tissues; however, it was shown that this phenomenon was unrelated to grading or lymph node metastasis. It might be more likely to invade if the antisense TE-13 cells exhibit a lower rate of apoptosis than the control TE-13 cells. PTEN and PDCD4 protein levels were higher in control tissues/cells compared to miR-21-overexpressing conditions whereas K-ras expression levels exhibited the opposite trend. The study suggested that miR-21 promotes ESCC development and spread partly through its regulation of PDCD4 and potential interaction with KRAS signaling (Liu L. et al., 2010).

Together, these studies in esophageal cancer highlight miR-21’s multifaceted roles in regulating cell adhesion, immune responses, core oncogenic pathways like PI3K/Akt, and potentially radiosensitivity, often through established targets like PTEN and PDCD4.

In esophageal squamous cell carcinoma (ESCC), the cell adhesion molecule CADM2 plays a significant role in tumor suppression. Low CADM2 expression is commonly observed in ESCC tissues, contributing to enhanced tumor growth and resistance to apoptosis. Overexpression of CADM2 in ESCC cells inhibits proliferation and induces cell death by blocking the Akt signaling pathway. This effect is further regulated by miR-21-5p, where its downregulation leads to decreased cell proliferation and increased apoptosis via the CADM2/Akt axis. Additionally, when CADM2 is knocked down, the anti-proliferative and pro-apoptotic effects of anti-miR-21-5p are attenuated, highlighting the crucial role of the miR-21-5p/CADM2-Akt pathway in regulating ESCC progression. These findings suggest that targeting this pathway could offer a novel therapeutic strategy for ESCC treatment. The researches on miR-21 and esophageal cancer are listed in Table 3.

**TABLE 3:** miR-21 and esophageal cancer.

Type of miR-21	Status of expression	Target	Model	Cell line type	Effect	Conclusion	Ref.
	Up	-	Human	-		The connection between miR-21 expression and CD3^+^ CD8^+^ T cell subgroup dysfunction may represent novel therapeutic targets	Wang et al. (2015b)
miR-21-5p	Up	PTEN	Human, *In vitro*	EC109, EC9706, THP-1	THP-1 macrophages generated by phorbol myristate acetate picked up EVs-miR-21-5p from EC109 or EC9706 cells and became M2 macrophages	Positive feedback between M2 polarity of macrophages and esophageal cancer cell EMT in the TME via miR-21-5p shuttling in tumor-derived EVs	Motoyama et al. (2010)
	Up	-	Human	-	miR-21 increased in endoscopic tissue biopsies	An alternative to histopathological diagnosis as our method provides a quick result following RNA isolation	Zhang et al. (2008)
	Up	-	Human, *In vitro*	HET-1A, ECA109, KYSE150, TE1	miR-21 were sensitive in the diagnosis of esophageal cancer	Esophageal cancer diagnosis relies on miR-21, which may be novel biomarkers	Yamanaka et al. (2012)
	Up	-	Human	-	miR-21 significantly overexpressed in ESCC cells	miR-21 may serve as non-invasive diagnostic biomarkers in patients with ESCC	Shiotani et al. (2012)
	Up	-	Human, *In vitro*	EC-109	miR-21 in EC-UT were significantly high	miR-21 in serum could serve as potential biomarkers for ESCC	Golestaneh et al. (2012)
	Up	PTEN	Human, *In vitro*	TE11	miR-21 promotes human esophageal cancer cell proliferation, migration, invasion, and cell cycle	A novel esophageal cancer treatment target maybe it	Jiang et al. (2011)
	Up	-	Human, *In vitro*	KYSE170	miR-21 was overexpressed	Plasma miR-21 in ESCC patients may indicate chemoresistance	Cao et al. (2012)
	Up	-	Human	-	MiR-21 levels were significantly raised in esophageal tissue and serum	Esophageal cancer may be diagnosed earlier with miR-21 and miR-375	Yang et al. (2014)
	Up	PDCD4, PTEN	*In vivo*	-	-	May be useful therapeutic targets in ESCC	Guo et al. (2013)
	Up	-	Human	-	miR-21 and miR-375 were of prognostic impact in ESCC	iR-21 was identified as an independent prognostic biomarker for DSS in patients with EAC	Ma et al. (2013)
	Up	CADM2	Human, *In vitro*	Het-1A, Eca109, TE-1, KYSE140, EC9706	MiR-21-5p downregulation caused cell death and growth inhibition	miR-21-5p/CADM2/Akt axis may treat ESCC differently	Kao et al. (2017)
	Up	PTEN, PDCD4, K-ras	Human, *In vitro*	TE13	mir-21 expression significantly increased in esophageal cancer tissues	Interaction with its PDCD4 and K-ras target genes may contribute to esophageal cancer development and metastasis	Liu et al. (2010b)
	Up	-	Human	-	increased levels of miR-21 in adenocarcinoma tissues	Esophageal cancer biomarkers may be diagnostic and predictive	Sha et al. (2014)
	Up	-	Human	-	miR-21 over-expressed in esophageal cancer	Could be used as novel molecular markers of esophageal carcinoma	Song et al. (2013b)
	Up	PDCD4	Human, *In vitro*, *In vivo*	Eca109	miR-21 was upregulated in Kazakh’s ESCC and that miR-21 played a negative role in regulating PDCD4	miR-21 may be a potential therapeutic target in management of ESCC	Eto et al. (2014)
	Up	-	Human	-	miR-21 showed a significant positive correlation with that of salivary miR-21	salivary miR-21 detection may substitute plasma testing for EC diagnosis	Shiotani et al. (2013)
	Up	TGF-β	Human, *In vitro*	EC9706	Overexpression of miR-21 in esophageal specimens	A novel oncogenic role of nicotine in human esophageal cancer	Stánitz et al. (2013)
	Up	PTEN	*In vitro*	K150, TE-1, EC-9706, EC-1, EC-109	Rawq01 also inhibited the PI3K-AKT pathway by upregulating PTEN expression via mir-21 inhibition	May help create chemosensitizing tumor therapy techniques by altering microRNA expression	Tu et al. (2014)
	Up	PTEN	Human, *In vitro*	HEEC, EC9706, EC-1, KYSE1170, KYSE410, KYSE180	Overexpression of miR-21 correlated with tumor status	May be an oncomiR that regulates PTEN and a novel prognostic factor for ESCC patients	Shi et al. (2013)
	Up	TPM1, SMAD, TGFβ1, FGF1, STAT3, STAG2, TIMP3, COL4A1	Human, *In vitro*	KYSE-30, OE-33, FLO-1	MiR-21 is primarily expressed in the SCC stroma and released from fibroblasts to affect SCC cell migration and invasion	May contribute to cellular crosstalk in the tumor microenvironment	Li et al. (2013b)
	Up	-	Human	-	miR-21 saliva supernatant were significantly upregulated in patients	Salivary miRNAs may identify esophageal cancer in high-risk locations	Chen et al. (2013)
	Up	-	Human	-	hsa-miR-21* was significantly increased in heavy drinking patients	May be useful biomarkers for high-risk population screening and detection	Cui et al. (2013)
	Up	PTEN	*In vitro*	TE-1	The inhibition of miR-21 significantly increased the cells’ radiosensitivity	To boost esophageal cancer radiosensitivity, miR-21 inhibition may be a novel treatment	Li et al. (2012)
	Up	PDCD4, TPM1	*In vivo*	-	Overexpression of miR-21 decreased PPP2R2A and PDCD4, their tumor-suppressor targets	miR-21 deregulation linked to inflammation explains how ZD promotes ESCC	Osawa et al. (2011)
	Up	-	Human	-	miR-21 was substantially greater in EC than controls	MiR-21 in saliva may be a new EC biomarker	Xu et al. (2012)
	Up	-	Human	-	-	-	Wang and Zhang (2012)
	Up	PTEN	Human, *In vitro*	Eca109	Overexpression of MiR-21 *in vitro* and ESCC increased cell growth	Might target PTEN at post-transcriptional level, and regulated the cancer invasion in Kazakh’s ESCC.	Takai et al. (2003)
	Up	-	Human	-	May help assess these individuals and identify those at risk of postsurgical recurrence	miR-21 correlate with tumor location and lymph node status	Kuramochi et al. (2001)
	Up	MAPK, TP53INP1, NFIB	Human, *In vitro*	TE-1, TE-3, TE-7, TE-8, HCE-4, HCE-7, SKGT-4, SKGT-5, Seg-1, Bic-1	-	-	Fukuhara et al. (2002)
	Up	-	Human	-	Noncancerous SCC tissue expresses miR-21 more	May help create prognostic biomarkers and new therapeutic targets and therapies	Chang et al. (2010)
	Up	-	Human, *In vitro*	TE1-15, Het-1A	miR-21 expression was substantially higher than in T1 or T2 cancers	May be involved in tumor growth and invasion	Li et al. (2018b)
	Up	p10	Human	-	Both cancers have 3- to 5-fold greater mir_21 expression than normal epithelium	May help identify Barrett esophagus patients at risk for adenocarcinoma	Rahir and Moser (2012)

Abbreviations: CADM2, Cell Adhesion Molecule 2; COL4A1, Collagen Type IV, Alpha 1 Chain; FGF1, Fibroblast Growth Factor 1; K-ras, Kirsten Rat Sarcoma Viral Oncogene Homolog; NFIB, Nuclear Factor I B; SMAD, sma and mad related protein; STAG2, Stromal Antigen 2; STAT3, Signal Transducer and Activator of Transcription 3; TGFβ1, Transforming Growth Factor Beta 1; TP53INP1, Tumor Protein P53 Inducible Nuclear Protein 1.

### MicroRNA- 21 and colon cancer

In order to learn more about colon cancer formation, invasion and migration, *in vitro* and *in vivo*, Wang et al. studied miR-21 along with its unidentified target genes. To modulate miR-21 levels, colon cancer cells were transfected using specific expression vectors: pmiRZip21 (designed to inhibit miR-21 function) or Leti3 (used as a control vector). When miR-21 was repressed *in vitro* and *in vivo*, TIMP-3 and RECK have been upregulated at the mRNA as well as protein levels significantly, while PCDH17 and BMPR-II have not been. The capacity of colon cancer cells to penetrate and move both *in vitro* and *in vivo* was significantly reduced after pmiRZip21 transfection. The ability of malignancies to penetrate and spread *in vitro* and *in vivo* may be restricted through upregulating TIMP-3 and RECK, which could serve to be a target site in anti-tumor treatment (Fridman et al., 2017). Similarly, Ni et al. investigated the molecular basis for Celastrol’s efficacy as an oncotherapy for colon cancer. Western blotting has been utilized to measure the concentrations of PCNA, PI3K, GSK3, and Akt, as well as phosphorylated Akt and GSK3. To find apoptosis, a method called flow cytometry was developed. They discovered that overexpressing a miR-21 mimic increased cell viability, suppressed apoptosis, lowered BCL-2 expression, increased BAX, and enhanced Caspase-3 activity, all of which were blocked by Celastrol. Additionally, Celastrol’s addition only partially prevented the PI3K/AKT/GSK-3 pathway from being activated by miR-21 mimic. Hence, Celastrol may reduce colon cancer cell growth by blocking the PI3K/AKT/GSK-3 pathway and miR-21 (Fridman et al., 2012). These initial studies establish miR-21’s role in promoting colon cancer cell invasion and survival, potentially through targets like *TIMP3*/*RECK* and modulation of the PI3K/Akt pathway, suggesting inhibitors like Celastrol could counteract its effects.

#### Regulation of prostaglandin signaling via 15-PGDH

More Cyclooxygenase-2 (COX-2)-derived Prostaglandin E2 (PGE2) was demonstrated to influence a number of cancer-related processes, such as apoptosis evasion, enhanced tumour angiogenesis, cell proliferation, and migration (Yu and Fu, 2006). PGE2 is easily transformed into its inactive state, and this catabolism may have an effect on the quantity of PGE2 in tissues. COX-2 is the enzyme that produces PGE2, and this enzyme has received the most attention for its contribution to the growth of cancer (Mosmann et al., 1997). It is now known that the enzyme 15-hydroxyprostaglandin dehydrogenase (15-PGDH; HPGD) is a crucial regulator of prostaglandin amounts since it breaks down PGE2 (Yang et al., 2015). The rate-limiting stage in prostaglandin catabolism, 15-PGDH inactivation, has been associated with PGE2 quantities in the colon (Kemp and Ronchese, 2001). The practically complete loss of 15-phosphogluconate dehydrogenase (PGDH) expression along with activity within human colorectal carcinomas when comparing to corresponding healthy tissue emphasizes the significance of PGDH during the initial stages of neoplastic formation (Yen et al., 2009). Poorer patient survival and more highly malignant phenotypes are associated with less 15-phosphogluconate dehydrogenase expression in gastric adenocarcinomas (Liang et al., 2015). 15-PGDH There are various regulatory domains in mRNA’s 3′untranslated region (UTR), namely, potential microRNA-binding sites and AU-rich patterns, which point to a function for post-transcriptional regulation in regulating the production of 15-PGDH (Zhang S. et al., 2018).

The research by Monteleone et al. shows that 15-PGDH can be controlled by miRNAs. These results show an inverse relationship between miR-21 and 15-PGDH in patients with Colorectal Cancer (CRC), with greater miR-21 quantities being linked to lower 15-PGDH expression. Because some locations within its 3′untranslated region (3′UTR) may directly regulate 15-PGDH, the presence of miR-21 within CRC cells lowers 15-PGDH levels while increasing levels of PGE2. Moreover, when epithelial growth factor (EGF) signaling raises miR-21 levels, miR-21 expression is downregulated. Consistent with this, reducing epidermal growth factor receptor (EGFR) signaling, which normally induces miR-21, complements the anti-proliferative effects of direct miR-21 inhibition in CRC cells. These findings demonstrate a unique method by which miR-21 controls 15-PGDH, as well as the potential role that aberrant miR-21 expression might have in the loss of 15-PGDH expression and the development of CRC (Wang, 2008). This work uncovers a novel link between miR-21, prostaglandin metabolism (via HPGD suppression), and EGFR signaling in CRC, providing a mechanism by which miR-21 can sustain pro-tumorigenic inflammatory signals. Inflammatory mediators are also tightly linked with miR-21 function in colon cancer.

#### Interaction with inflammatory cytokines (TNF-α, IL-6)

Inflammation promotes colorectal cancer. Cancer tissue has higher levels of the powerful inflammatory cytokine tumor necrosis factor alpha (TNF-α) (Aandahl et al., 2008) and promotes cancer (Liu J. et al., 2013). A number of malignancies have high TNF- levels associated with metastasis, including colorectal cancer (Rahmati et al., 2021). Type II transmembrane protein TNF- is synthesized in cells and exists as stable homo-trimers (Li P. et al., 2013). TNF- conversion enzyme proteolytically cleaves TNF- to produce the active soluble homo-trimeric cytokine (TACE). A series of cellular events are triggered when the cytokine binds to TNF- receptors (TNFR). Intriguingly, Cottonham et al. (Tanaka et al., 2013) discovered in colorectal cancer cells that miR-21 is positively controlled by TNF- and TGF-, and that these cells had increased motility and invasiveness in an organoid model. According to Qiu et al. (Samiei et al., 2019) Chen et al. (Samiei et al., 2022), in a diabetic nephropathy model of rat, it was shown that pulling down miR-21 expression decreased TNF- release. Furthermore, he discovered a link among miR-21 as well as TNF- in oral cancer cells, in which it managed proliferative and fatal behavior. As a result, many studies suggest that miR-21 and TNF may interact in an autocrine and paracrine manner.

Trine Mller et al. were interested in the functions of miR-21 in cell viability and TNF- in necrosis at the invasive front of colon tumors. To co-stain miR-21, TNF- mRNA, and cytokeratin, the researchers created an automated method utilizing frozen colon cancer tissue samples (n = 4). TNF- mRNA expression has been only seen in the four instances’ most aggressive cancer cells. Utilizing confocal slide scanning microscopy-produced digital slides, the expression of TNF-mRNA and miR-21 was studied. In developing cancer cells, expression of miR-21 has been observed within both concordant as well as discordant patterns. When comparing to TNF- mRNA, miR-21 was found to be more abundant in cancer cells. Even though miR-21 is unrelated to the expression of mRNA in the pro-inflammatory cytokine TNF-, they conclude that TNF- and miR-21 simultaneously assist to tumor growth at the tumor’s invasive front in colon cancers. They suggest that miR-21 protects cancer cells and fibroblasts from autocrine and paracrine TNF-induced cell death (Yang et al., 2015). Studies also indicated that there is a mutual relationship between interleukins and miR-21 in the context of cancer development. Saroor and colleagues indicated that immune cells which produced IL-6 in colorectal cancer induced tumor progression and in the presence of this interleukin, cancer cells produced miR-21 and miR-29b to further induce immune cell IL6 production (Bertrand et al., 2014). Many genes linked to cancer have been suggested as potential targets for the miR-21 microRNA. They consist of cell division cycle 25A, tropomyosin 1, protein sprouty homologues one and 2, programmed cell death protein 4 (PDCD4), and protein sprouty homologues 2 (Wang J. et al., 2015). The maintenance of T cell effects and the control of protracted inflammatory processes are additional functions of miR-21 (de la Chapelle and Jazdzewski, 2011). These studies collectively implicate miR-21 within the complex inflammatory milieu of colon cancer, linking it to key cytokines like TNF-α and IL-6 and suggesting it plays a role in both promoting growth and protecting tumor cells from inflammation-induced death, particularly at the invasive margin. This connection between inflammation and miR-21 is particularly relevant in colitis-associated cancer.

#### Role in colitis-associated cancer (CAC)

The researchers Shi et al. set out to learn more about miR-21’s role in colitis-related colon cancer (CAC) by looking into its molecular underpinnings. MiR-21 expression was investigated in the tumors of 62 Chinese, 37 Japanese, and Austrian individuals with CRC who just had colitis-related neoplasms. Many *in vitro* and *in vivo*, as well as clinical methods were used in investigating the biological roles of miR-21. Tumors from 62 CRC patients, 22 CAC patients, and a mouse model of CAC all showed a substantial increase in miR-21. Interleukin (IL) 6, IL-17A, and IL-23, as well as IL-21 production was reduced, and tumour sizes were decreased in miR-21-knockout mice following treatment with azoxymethane and dextran sulphate sodium. Tumor cell proliferation and E-cadherin expression were both suppressed in CAC mouse tumors lacking miR-21, but -catenin and SOX9 levels were increased. Moreover, in miR-21 knockout mice, expression of its target gene *PDCD4* was increased, which unexpectedly correlated with enhanced NF-κB activation, highlighting complex downstream effects in the CAC model. In CAC mice, miR-21 removal boosted tumor cell mortality, which was followed by a decline in STAT3 and Bcl-2 activation. Their results indicate for the first time that inhibiting miR-21 is an effective way to reduce CAC levels (Kar et al., 2015). This genetic knockout study provides strong *in vivo* evidence for miR-21’s causal role in promoting colitis-associated colorectal cancer, linking its function to inflammatory cytokine production, proliferation control via β-catenin/SOX9, target gene PDCD4, and suppression of apoptosis via STAT3/Bcl-2. Finally, miR-21 plays a critical role in chemoresistance and stem cell characteristics in colon cancer. These findings highlight the PTEN/PI3K/Akt axis as a critical downstream pathway modulated by miR-21 in gastric cancer, suggesting that interventions targeting miR-21 (like curcumin) might restore PTEN function and inhibit tumor growth.

#### Contribution to chemoresistance and cancer stem cells (CSCs)

One of the main factors contributing to the failure of cancer therapy is drug resistance to previously successful cancer treatments. Although the existence of cancer stem cells (CSCs) was theorized as a contributing factor, this is still not fully understood. Colon cancer cells’ stemness is encouraged by miR-21 (Zhang et al., 2010). Additionally, research has shown that 5-Fluorouracil (5-FU)’s therapeutic potency is markedly diminished by miR-21 overexpression (Si et al., 2007). Since the use of the EGFR inhibitor Cetuximab, it has been discovered that EGFR is related to the miR-21 control (mAb to EGFR), and it's been found to reduce miR-21 production (Hong et al., 2013). In contrast to miR-21, miR-145 has been a tumour suppressor that is regulated by p53 and has been linked to a variety of cancers, including colorectal cancer (Yan-nan et al., 2014). By repressing several pluripotent genes, such as OCT4, SOX2, and KLF4, it controls stem cell renewal and pluripotency (Wu et al., 2016).

The research conducted by Yu et al. sought to identify the molecular basis for miR-21’s regulatory role within colon cancer stem cells. The pCMV/miR-145 expression plasmid or transfection of mature, antagonistic miRs were used to regulate the levels of miR-21 and miR-145. Researchers discovered that miR-21 was noticeably increased and miR-145 was noticeably downregulated in CSC-enriched chemoresistant (CR) colon cancer cells. They showed that miR-145s forced expression in colon cancer cells causes the exact opposite effect from that brought on by upregulating the miR-21, considerably inhibiting the growth of CSCs and tumours. Moreover, SCID mice treated with mature antagomir-21 or miR-145 (anti-sense miR-21) exhibit markedly reduced colon cancer cell xenograft development. Because the expression of CD44, Sox-2 and -catenin was lowered and also CK-20 was activated, this shows that antagomir-21 or miR-145 therapy reduces CSC proliferation as well as encourages differentiation. More proof is provided by *in vitro* studies showing a negative regulatory relationship between miR-145 and miR-21. It seems that k-Ras is important for the control of this process. This is shown by the fact that within CR colon cancer cells, the absence of k-Ras results in a rise in miR-145 production, a decrease in miR-21 production, and an interruption of the collaboration among miR-21 and miR-145, which would typically result in a negative feedback loop. Current research demonstrates the importance of networks that the microRNAs miR-145 and miR-21 are a part in regulating colon cancer chemoresistance as well as the proliferation or/and diversification of cancer stem cells (CSCs) (Hummel et al., 2011). Highlighting the complexity of miRNA networks, these findings demonstrate a reciprocal negative regulation between the oncomiR miR-21 and the tumor suppressor miR-145, modulated by KRAS signaling, which collectively governs CSC properties and chemoresistance in colon cancer. MiR-21 plays a significant role in colon cancer progression, including tumor formation, invasion, and migration. Furthermore, the involvement of COX-2-derived PGE2 in cancer progression underscores its role in colon cancer. PGE2 influences key processes such as cell proliferation and migration, and its catabolism by 15-PGDH regulates its levels in the tumor microenvironment. Loss of 15-PGDH has been linked to elevated PGE2 levels, contributing to colon cancer progression. These insights highlight the potential for targeting miR-21 and related pathways for therapeutic intervention in colon cancer. Table 4 contains a collection of research on miR-21 and colon cancer.

**TABLE 4:** miR-21 and colon cancer.

Type of miR-21	Status of expression	Target	Model	Cell line types	Effect	Conclusion	Ref.
miR-21-3p, miR-21-5p	Up	-	Human, *In vivo*	-	-	As a potent diagnostic tool for CC determination	Yang et al. (2013b)
miR-21-3p	Up	-	Human	-	The early T-stages were related with hsa-miR-21-3p	An innovative approach to CRC diagnosis and therapy	Wen et al. (2015)
	Up	PDCD4	*In vitro*	-	miR-21 overexpression associated with CC	Presents a new theoretical foundation for CC incidence and development	Song et al. (2021)
	Up	-	Human	-	-	Enhance understanding of local tumor molecular differences	Crawford et al. (2020)
	Up (stage dependent)	-	Human	-	Levels of miR’s −21increased in non-vesicular to extracellular vesicular	Detect early-stage CRC in a multiethnic, untested population	Yao et al. (2019b)
	Up	-	Human	-	miR-21 were significantly increased	It may be a new colon and breast cancer biomarker, pharmacological target, or gene target	Zhang et al. (2018c)
	Up	PI3K	*In vitro*	HCT-116	miR-21 mimic overexpression could enhance the cell viability	Celastrol may reduce colon cancer cell growth by affecting miR-21 and the PI3K/AKT/GSK-3β pathway	Fridman et al. (2012)
	Up	TIMP-3, RECK	*In vitro*, *In vivo*	SW480, SW620, HCT-116, LoVo	In colon cancer cells transfected with pmiRZip21 miR-21 expression decreased	potential as a possible target site in anti-tumour therapy	Fridman et al. (2017)
	Up (stage dependent)	-	Human	-	*miR-21* in stage II was significantly different from stage IV	Can be a good molecular marker for classification of the stages of colon cancer	Wang et al. (2018c)
	Up	PDCD4, PTEN	*In vitro*	HCT-116, CoN ATCC CRL-1790	bPGN inhibits miR-21 and its target genes such PDCD4 and PTEN, supporting on-target inhibition	As a molecular probe or therapeutic treatment for miRNA-dependent illnesses	Komatsu et al. (2016)
	Up	-	*In vivo*	-		Noninvasive biomarkers can be used as indicators to screen early colon cancer progression	Lv et al. (2016)
	Up	-	Human	-	miR-21 was more often seen in cancer cells than TNF-α mRNA	miR-21 may shield fibroblasts and cancer cells from TNF-α-induced cell death	Rahmati et al. (2021)
	Up	15-PGDH, PTEN	Human, *In* *vitro*	HeLa, HCT-15, LS174T, HT-29, Caco2, HCT-116	In CRC patients, miR-21 levels are inversely associated to 15-PGDH expression	May reduce 15-PGDH expression and enhance CRC advancement by increasing PGE2	Mosmann et al. (1997)
	Up	-	Human, *In vitro*	SW480	Patients with high miR-21 expression had a significantly shorter survival time	May be involved in the regulation of colon cancer cell proliferation	Fong et al. (2016)
	Up (stage dependent)	-	Human	-	Some miR-21-positive TBCs also showed laminin-5γ2 positivity	Digital slides from confocal slide scanning microscopy revealed miR-21 expression in TBCs	Winther et al. (2015)
	Up	-	Human	-	miR-21 associated with TNM classification and clinical staging of adenocarcinoma	Possible novel, non-invasive marker for early CRC diagnosis, screening, and prognosis	Hezova et al. (2015)
	Up	-	Human	-	miR-221 was significantly higher in rectal cancer than in colonic cancer	-	Stánitz et al. (2015)
miR-21-5p	Down	Tiam1	Human, *In vitro*, *In vivo*	FIHC, HCT-116, HT-29, SW480	miR-21-5p was expressed at a low level	May offer novel colon cancer treatment targets	Liu et al. (2014)
	Up	IL6R	Human	-	Restricted EPC behavior, and miR-21 removal eliminated the decreased proliferation	Provide novel treatment targets	Ye et al. (2014)
	Up	Wnt	*In vitro*, *In vivo*	SW480	MiR-21 was downregulated by JQ-1	Mechanism of JQ-1 action is associated with its regulation of Wnt/β-catenin signaling and miR-21 levels	Zhang et al. (2014)
	Up	-	Human	-	Increased expression in stool of CC patients	-	Weng et al. (2013)
	Up	ITGβ4, PDCD4	*In vitro*	HCT116	Berberine’s anti-cancer actions and apoptosis involve miR-21	-	Li et al. (2013c)
	Up	-	Human	-	evaluated in the normal mucosa adjacent to tumor	Can be used as tissue biomarkers also to the tumor-adjacent mucosa	Nouraee et al. (2013)
miR-21-5p	Up (stage dependent)	-	Human	-	Was detected in stage pT1	Could support risk assessment in stage T1 tumors	Xie et al. (2013)
	Up	ING3	Human, *In vitro*	SW480, HCT-116, FHC	CASC7 suppressed miR-21/ING3-mediated colon cancer cell growth and migration	may be considered as a novel diagnostic marker of CRC	Huang et al. (2013)
	Up	-	Human, *In vitro*	DLD-1, HCT116, HT29, HT55, SW837, VACO4S		May offer translational opportunities	Alder et al. (2012)
	Up	-	*In vitro*	RKO, HCT 116	Practical doses of SFN significantly downregulated oncogenic miR-21	Is a promising approach for delaying and/or preventing CRC	Xie et al. (2012)
	Up	-	Human	-	A high miR-21 expression was associated with reduced progression-free survival (PFS)	May predict chemotherapeutic success and survival in unresectable metastatic CC patients	Hamano et al. (2011)
	Up	PTGS2	Human	-	MIR21 expression was associated with CRC-specific mortality in PTGS2-high tumors	Complex immunity and inflammatory roles in tumor progression	Ma et al. (2011)
	Up	TNFα	*In vitro*, *In vivo*	HCT-116, HCT-8	A DHA-rich diet reduced miR-21 expression	A novel mechanism for anti-cancer action of DHA	Hu et al. (2011)
	Up	PTEN	Human	-	-	may distinguish recurred from non-recurred patients	Mathé et al. (2009)
	Up	-	Human, *In vitro*	CACO2, HT29, HCT116	In colon cancer cell lines, high folate boosted miR-21 expression	As a colorectal cancer biomarker	Mori et al. (2009)
	Up	-	*In vitro*, *In vivo*	SW-48	-	-	Feber et al. (2008)
	Up	PTEN	Human	-	The tumor-associated stroma with the greatest rise in miR-21 from adenoma to adenocarcinoma	May play a role in the development of colon cancer	Wang et al. (2019b)
	Up	-	Human	-	Tumor MIR21 expression was inversely associated with densities of CD3(+) and CD45RO(+) cells	As a potential target for immunotherapy and prevention in colorectal cancer	Ni et al. (2019)
	Up	PDCD4	*In vitro*	RKO	The apoptosis ratio increased in miR-21 knockout cells	MiR-21 can mediate the drug resistance to 5-FU	Dixon et al. (2013)
	Up	PTEN	*In vitro*	HCT-116, HT29, SW620, FHC	miR-21 increased the expression of its target molecule PTEN	Therapeutic strategies against clinical colon carcinoma	Sheng et al. (1998)
	Up	RECK	*In vitro*	SW480, LS174	Blockage of RAGE with anti-RAGE antibody suppresses S100P induction of miR-21	S100P induces miR-21 expression via ERK and is inhibited by U0126	Rozic et al. (2001)
	Up	PDCD4	Human, *In vivo*	-	miR-21 increased the expression of its target gene PDCD4	Novel evidence for miR-21 blockade to be a key strategy in reducing CAC.	Kar et al. (2015)
	Up	PDCD4	*In vitro*, *In vivo*	HCT-116, HT-29	miR-21 negatively regulates miR-145 and *vice versa*	-	Hummel et al. (2011)
	Up	RASA1	*In vitro*, *In vivo*	RKO, HCT116, colo205, SW480, HT29, Caco2	RASA1 is a target gene of miR-21	Promotes malignant behaviors of RKO cells through regulation of RASA1 expression	Tai et al. (2006)
	Up	-	Human	-	Defined the CRC metastatic signature	microRNA can distinguish colorectal lymph node and liver	Tai et al. (2002)
	Up	hMSH2 TP, DPD	*In vitro*	HT-29, HT-29/5-FU	Significantly inhibited apoptosis, enhanced cell proliferation, invasion	May contribute to colon cancer cells’ 5-FU resistance	Myung et al. (2006)
	Up	-	Human	-	-	It may help counsel and personalize disease management	Backlund et al. (2005)
	Up	ITGβ4, PDCD4, PTEN	Human, *in vitro*	Caco-2, DLD-1, HT-29, SW620, HCT116, Colo 205, RKO	miR-21 is a key player in oncogenic EMT	Could be used to predict CRC metastasis	Tatsuwaki et al. (2010)
	Up	-	Human	-	Associated with poor therapeutic outcome	May also identify adjuvant chemotherapy candidates	Moore et al. (2011)
	Up	PTEN	*In vitro*, *In vivo*	HCT-116, HT-29, SW620	Increased miR-21 expression	May treat chemotherapy-resistant colorectal cancer	Khan et al. (2016)
	Up	PDCD4	*In vitro*, *In vivo*	SW480, Caco-2, HT29	AR inhibition downregulates miR-21 to suppress growth	-	Monteleone et al. (2019)
	Up	-	Human	-	-	-	Old (1985)
	Up	PTEN PDCD4, βR2	*In vitro*	HCT-116, HT-29	Antisense miR-21 induced differentiation	-	Carswell et al. (1975)
	Up	-	Human	-	Mostly fibroblast-like cells showed miR-21 signal	miR-21 selection of high-risk stage II colon cancer patients is warranted	Arnott et al. (2002)
	Up	PTEN	*In vitro*, *In vivo*	SW480, Caco-2, HCT116, HT29	miR-21 expression, suggesting FOXO3a inactivates AP-1 to shut down miR-21	A novel role of AR in the regulation of miR-21	Balkwill (2002)
	Up	PTEN	Human, *In vitro*	LoVo, SW480, caco2	Overexpression of PRL-3 in colon cancer cells activated STAT3 to produce miR-21	PRL-3-induced miR-21 promotes colon cancer growth and invasion	Guadagni et al. (2007)
	Up	-	Human, *In vitro*	DLD-1	Overexpression of miR-21 in CRC samples and advanced disease	CRC cells with increased expression of miR-21 have higher migration ability	Grimm et al. (2011)
	Up	Spry2, PTEN	*In vitro*	HCT116	mir-21 induced upregulation of Spry2 and PTEN	A potential strategy for colon cancer treatment	Ham et al. (2016)
	Up	PDCD4, PTEN	*In vivo*	-	In colon, miR-21 expression rises with age	Could be used as biomarker	Qu et al. (2017)
	Up	TGFβR2	*In vitro*, *In vivo*	HCT-116, HT-29	downregulation of miR-21 enhances luciferase-TGFβR2-3′ UTR activity	Contributes to stemness regulation via altering TGFβR2 signaling	Yu et al. (2015)
	Up	PTEN, PPP2CA, SOCS1	Human, *In vitro*, *In vivo*	HCT-116, DLD1, SW480	NTR1 activation stimulates expression of miR-21	Correlate with tumor stage	Qiu et al. (2018)
	Up	-	*In vivo*	-	linked to canonical oncogenic signaling pathways	-	Chen et al. (2018b)
	Up	-	Human	-	Disease-free survival was considerably shorter with miR-21 expression	miR-21 is primarily a stromal microRNA	Møller et al. (2019)
	Up	-	Human	-	Patients showed higher expression of miR-21	Could be used as biomarker	Dehghan et al. (2019)
	Up	Cdc25A	*In vitro*	RKO, DLD1	miR-21 in modulating cell cycle progression	A potential explanation of miR-21 in tumorigenesis	Wang et al. (2009b)
	Up	Cdc25A	*In vitro*	HCT116	-	-	Asangani et al. (2008)
	Up	SPRY2	*In vitro*	SW480, SW620	-	MiR-21 specifically targets and downregulates SPRY2 to promote cellular protrusions	Meng et al. (2007)
	Up	-	Human	-	miR-21 to be expressed at high levels in colonic carcinoma cells	miR-21 expression is associated with poor survival and poor therapeutic outcome	Zhu et al. (2007)
	Up	-	*In vitro*	HT-29, HCT-116	miR-21 that is associated with anti-apoptotic functions characterizing malignant cells	-	Sayed et al. (2008)

15-PGDH, Prostaglandin Dehydrogenase 15; Cdc25A, Cell Division Cycle 25A; DPD, dihydropyrimidine dehydrogenase; IL6R, Interleukin 6 Receptor; ING3, Inhibitor of Growth Family Member 3; ITGβ4, Integrin Beta 4; PI3K, Phosphoinositide 3-Kinase; PPP2CA, Protein Phosphatase 2 Catalytic Subunit Alpha; PTGS2, Prostaglandin-Endoperoxide Synthase 2 (Cyclooxygenase-2); PDCD4, Programmed Cell Death 4; RECK, reversion inducing cysteine rich protein with kazal motifs; RASA1, Ras P21 Protein Activator 1; SPRY2, Sprouty RTK, Signaling Antagonist 2; SOCS1, Suppressor of Cytokine Signaling 1; TGFβR2, Transforming Growth Factor Beta Receptor 2; Tiam1, T-Cell Invasive Antigen 1; TNFα, tumor necrosis factor alpha; TP, thymidine phosphorylase; Wnt, Wingless-Type MMTV, integration site family member; βR2, Beta Receptor 2; hMSH2, MutS Homolog 2 (Human).

### Exosomal miR-21 and gastrointestinal cancers

Beyond its intracellular functions, miR-21 is actively packaged into exosomes—small extracellular vesicles (EVs) secreted by various cell types, including cancer cells and stromal cells within the tumor microenvironment (TME). These exosomes serve as potent vehicles for intercellular communication, transferring their cargo, including miRNAs like miR-21, to recipient cells (Shi et al., 2016). This transfer allows exosomal miR-21 to exert profound effects on recipient cell behavior, thereby driving key aspects of GI cancer progression. Key roles attributed to exosomal miR-21 include modulating the TME (e.g., influencing macrophage polarization or fibroblast activity), promoting metastasis through processes like EMT, contributing to chemoresistance, and serving as a circulating biomarker for diagnosis and prognosis (Nautiyal et al., 2012; Martínez-Gutierrez et al., 2022; Han Z. et al., 2021). This section will explore the evidence supporting these diverse functions of exosomal miR-21 in GI cancers.

Exosomes are tiny membrane vesicles (30–100 nm) which transport lipids, proteins, messenger RNAs (mRNAs), along with microRNAs (miRNAs) from 1 cell to another (Shi et al., 2016). Research on the biological importance of MV function has increased significantly recently (Yu et al., 2012). It has been shown that some cell types, such as cancer cells, release miRNAs into different physiological fluids via exosomes (Valeri et al., 2010). MiR-21, which is formed from exosomes and was connected to colon cancer cell invasion and proliferation, is studied via researchers Sun et al. to comprehend its mechanisms and how it functions. Human colonic adenocarcinoma cell lines’ culture media were ultracentrifuged to separate exosomes, and several biochemical methods, including Western blotting, real-time quantitative polymerase chain reaction, and electron transmission microscopy, were used to measure the expression levels of miR-21. Afterward, using the colon cancer cell lines T84, LS174, and HT29 along with the normal colon epithelial cell line CRL 1831, they carried out *in vitro* functional assays (proliferation, invasion) and mechanistic studies (Western blot for target genes). Thus, Colon cancer cells expressed significantly more miR-21 exosomes than normal human colon epithelial cells. Genes engaged in cell invasion, and proliferation as well as extracellular matrix synthesis were upregulated when colon cancer cell lines received treatment *in vitro* with miR-21 mimics or isolated exosomes. Treatment with these exosomes decreased protein levels of miR-21 targets PDCD4, TPM1, and PTEN in recipient cells; notably, direct siRNA silencing of PDCD4 mimicked the pro-invasive and chemoresistance-inducing effects of exosomal miR-21 transfer. Their findings imply that blocking exosomes, and especially those that contain miR-21, might be an effective new way to treat colorectal cancer (Nautiyal et al., 2012). This foundational study demonstrates that colon cancer cells actively release miR-21 via exosomes, which can functionally transfer oncogenic potential (proliferation, invasion, chemoresistance) to recipient cells, partly by suppressing targets like PDCD4.

#### Mechanisms and role in metastasis (PDAC, ESCC)

**FIGURE 3:**
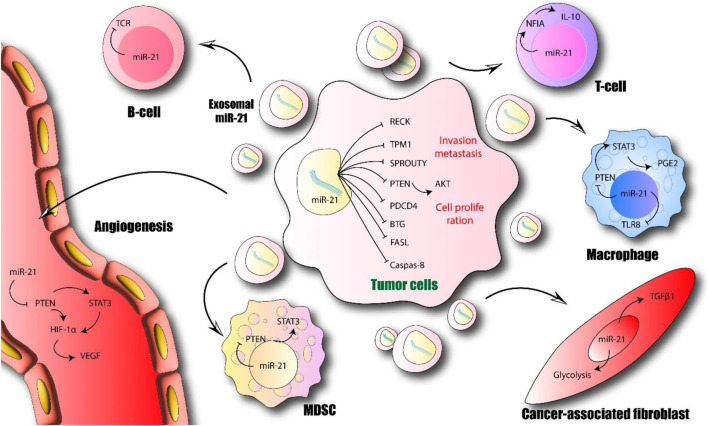
Role of exosomal miR-21 in progression of cancer. Exosomal miR-21 regulates the tumor microenvironment via different signaling pathways in endothelial cells, tumor cells, cancer-associated fibroblasts, and immune cells. AKT, RAC-alpha serine/threonine-protein kinase; BTG, B-cell translocation gene; FASL, Fas ligand; NF1A, nuclear factor 1 A-type; PDCD4, programmed cell death 4; PTEN, phosphatase and tensin homolog; HIF-1α, hypoxia-inducible factor 1α; RECK, reversion-inducing-cysteine-rich protein with Kazal motifs; STAT3, signal transducer and activator of transcription 3; TCR: T-cell receptor; PGE2, prostaglandin E2; TLR8; Toll-like receptor 8; TPM1, tropomyosin 1; VEGF, vascular endothelial growth factor (author-created).

Exosome-mediated intercellular communication significantly controls cancer cell activity (Sachdeva et al., 2009; Bandrés et al., 2006). Particularly exosomal microRNAs (miRNAs) encourage tumour growth by inhibiting their natural regulators (Bandrés et al., 2006). miR-21 plays a range of roles in cancer, such as those connected to metastasis. A prometastatic phenotype is brought on by the transfer of miR-21 from hypoxic oral squamous cell carcinoma exosomes to normoxic cells (Yu et al., 2015) Exosomal miR-21 was shown to enhance the invasion as well as migration of esophageal cancer cells through inhibiting programmed cell death (Figure 3) (Martínez-Gutierrez et al., 2022). MiR-21 expression in exosomes generated by pancreatic stellate cells was shown to be greater in patients with pancreatic ductal adenocarcinoma (PSCs) (PDAC) (Martínez-Gutierrez et al., 2022). The goal of Ma et al.’s study was to determine how exosomal miR 21 affects PDAC cells’ migration and look into the potential molecular mechanism underlying this link. In people with pancreatic adenocarcinoma, increased miR 21 levels have been related to a weak prognosis, according to analyses of the Ras/ERK signalling pathway as well as the Cancer Genome Atlas database. *In vitro* migration and invasion assays on PDAC cells demonstrated that internalization of PSC-derived exosomes containing high miR-21 levels stimulated cell motility, raised matrix metalloproteinase 2/9 activity and induced an epithelial-to-mesenchymal transition (EMT). Exosomal miR-21 also raised the phosphorylation of Akt and ERK1/2 (measured by Western blot) within PDAC cells. Our findings suggest that exosomal miR 21 produced by PSCs may increase Ras/ERK signalling activity, speed up PDAC cell motility, and trigger EMT. As a result, miR 21 might be a novel therapy target and a reason why certain pancreatic cancer patients have a bad prognosis (Han Z. et al., 2021).

There is evidence that exosome-formed miRNAs made by tumour cells can enter recipient cells and function biologically there. The miR-21 gene was present in the conditioned medium of esophageal cancer cells. Additionally, esophageal cancer cells co-cultured with fibroblasts showed a significant increase in miR-21, facilitating the transfer of miRNAs that shut down exosomes (Eslamizadeh et al., 2021). Liao et al. looked into the significance of the exosome-shuttling miR-21 in the emergence of esophageal cancer in light of the growing body of evidence indicating exosomes may well be employed to transmit genetic data across cells. Using fluorescence microscopy in co-culture experiments, it was discovered that exosomes enter recipient cells. Furthermore, pharmacological inhibition showed that neutral sphingomyelinase 2 (nSMase2) was required for the uptake of exosomal contents like Cy3-labeled miR-21 mimics. Functionally, exosomal miR-21 transfer increased recipient cell motility and invasion, which was mechanistically linked to the suppression of its target PDCD4 and activation of the JNK signaling pathway. Based on their analysis of plasma samples from a cohort of esophageal cancer patients and healthy controls, they discovered that miR-21 has been much greater within plasma from patients with esophageal cancer, indicating a strong risk link with this disease. Their results showed a strong association among exosome-shunting miR-21 as well as esophageal cancer progression and recurrence. As a result, miR-21, which exosomes may carry, has the ability to predict how well esophageal cancer patients would fare (Martínez-Gutierrez et al., 2022). These studies in PDAC and ESCC extend the functional role of exosomal miR-21 to mediating pro-metastatic communication between different cell types in the TME (stromal cells to cancer cells) and promoting migration/invasion through pathways involving Ras/ERK/Akt and *PDCD4*/JNK, further linking exosomal miR-21 to poor prognosis. Beyond its functional roles, exosomal miR-21 holds promise as a biomarker.

#### Exosomal miR-21 as a diagnostic and prognostic biomarker (GC, PM)

MicroRNA (miRNA) analysis in exosomes provides valuable data for early detection of cancer without invasive biopsies. Although miRNAs may be useful indicators in the clinic, their modest quantity in exosomes frequently limits their use. Exosomal miR-21 is detected with high sensitivity by a biosensor developed by Xia et al. that uses dual-signal amplification (miR-21). It creates a DNA-RNA heteroduplex in the existence of a cognate target using the biotin-modified capture probe (Cp) and acts as a substrate for such a nuclease that only recognises duplexes (DSN). The DSN performs enzymatic digestion of the Cps, causing the release of numerous DNA catalysts that support the first signal amplification. The DNA catalyst in the supernatant starts a strand displacement reaction employing a nicking-assisted reactant recycling approach to carry out the second stage of signal amplification after magnetic separation. Their biosensor, which employs the dual-signal amplification principle, can detect miR-21 with such a linear range between 0.5 and 100 fM as well as a detection limit of 0.34 fM. Exosomal miR-21 beats serum carcinoembryonic antigen in detecting patients having gastric cancer (GC) as well as patients having precancerous lesions (PC), according to the receiver operating characteristic curve created throughout clinical sample analysis (area under the curve: 0.89 versus 0.74, n = 40). Using a confusion matrix, the proposed biosensor could distinguish between patients who have PC or GC lesions versus normal donors with an accuracy of 83.9%. The suggested biosensor can also distinguish between individuals with GC who have or have not yet developed metastases. DNA-based biosensor-enabled cancer diagnostics may find new uses thanks to their technology (Shiosaki et al., 2020).

Peritoneal metastasis (PM) patients have a poor prognosis despite it being the location where gastric cancer metastasizes most frequently. It may be difficult to recognize peritoneal lesions, making it tough to decide whether to change the course of treatment. Peritoneal metastases are challenging to detect with Computed Tomography (CT) scans because they are frequently too tiny to accurately assess for size reduction, even with advanced technology. Accurate molecular indicators are necessary to determine the volume of tumor inside the peritoneal cavity.

In order to find new markers that indicate the presence of tumors in the peritoneum, Ohzawa et al. studied the exosomal miRNA profile within peritoneal fluids. Exosomes from gastric cancer patients with either microscopic (P0CY1) or macroscopic (P1) peritoneal metastases (PM) were collected, as well as their miRNA expression profiles were carefully examined. TaqMan Advanced miRNA Assays were then used to confirm the expression of putative miRNAs in all 58 samples. After obtaining the peritoneal fluid from 11 individuals who have PM and 14 individuals who do not have PM, they performed a thorough analysis of exosomal miRNA. In PM (+) samples, they found 11 dysregulated miRNAs. The validation study’s findings revealed a connection between the peritoneal cancer index and four miRNAs, whose expression levels were noticeably higher there in 12 p.m. (+) samples (miR-21-5p, miR-223-3p, and miR-92a-3p, as well as miR-342-3p). individuals who have PM (+) samples, in contrast, showed downregulation of such entire miR-29 family. Furthermore, miR-29 at gastrectomy showed significant decrease in 6 individuals who have peritoneal recurrence among 24 patients with pT4 tumors (P = 0.012) due to significant changes in miR-29b-3p. PM management may benefit from using the miRNA expression pattern in peritoneal exosomes, which is highly correlated with the amount of tumor in the peritoneal cavity (Kundaktepe et al., 2020).

Collectively, these biomarker studies highlight the potential of measuring exosomal miR-21 levels in various bodily fluids (plasma, peritoneal fluid) using sensitive detection methods as a non-invasive tool for early diagnosis, monitoring tumor burden (e.g., in peritoneal metastasis), and potentially predicting prognosis in GI cancers.

These findings underscore the pivotal role of exosomal miR-21 in the metastatic spread and resistance of gastrointestinal cancers, suggesting its potential as a therapeutic target. Table 5 lists exosomal miR-21 and GI cancers.

**TABLE 5:** Exosomal miR-21 and GI cancers.

Type of cancer	Status of expression	Target	Model	Cell line types	Effect	Conclusion	Ref.
Colon cancer	Up	TPM1, PTEN, PDCD4	*In vitro*	HT29, T84, LS174	Induced resistance against 5-FU	May represent a novel approach for treatment	Nautiyal et al. (2012)
	Up	-	Human, *in vitro*	FHC, HCT116, HT-29, RKO, SW48, SW480	Significant in primary CRC patients, especially early-stage illness	Promising biomarkers for non-invasive diagnosis of the disease	Dehghan et al. (2019)
Gastric cancer	Up	-	Human	-	-	-	Shiosaki et al. (2020)
	Up	-	Human	-	-	Useful biomarker in the treatment of PM.	Kundaktepe et al. (2020)
	Up	PDCD4	Human	-	MiR-21 and programmed cell death protein 4 mRNA expression correlated negatively	Useful biomarker in the treatment	Matarlo et al. (2019)
	Up	SMAD7	Human, *In vivo*, *In vitro*	MGC803, BGC823, MKN45, HGC27, SGC7901, GES-1, HMrSV5	Promote tumor peritoneal metastasis	Exosomal miR-21-5p may be a novel therapeutic target for GC peritoneal metastasis	Li et al. (2018c)
	Up	-	*In vitro*	MKN45, SGC7901, NCI-N87, AGS, GES-1	most abundant sequences among	may make a little contribution to the understanding of exosomal RNA composition	You et al. (2019)
	Up	-	Human *In vitro*	OCUM-2M, OCUM-2MD3	Associated with serosal invasion in GC	May serve as biomarkers of peritoneal recurrence after curative GC resection	Knudsen et al. (2018)
	Up	-	Human	-	-	-	Sabry et al. (2019)
Pancreatic cancer	Up	-	Human	-	-	As PC diagnosis and treatment biomarkers and targets	Orosz et al. (2018)
	Up	Ras	Human, *In vitro*	PANC-1, MIAPaCa-2	Promoted cell migration, induced EMT and increased matrix metalloproteinase-2/9 activity	May be a potential cause of poor prognosis in patients	Bandrés et al. (2006)
	Up	-	Human	-	Candidate prognostic factor for the overall survival	Useful marker	He et al. (2018)
	Up	-	Human	-	-	-	Wang et al. (2018d)
	Up	-	Human	-	Increased in exosomes	As diagnosis and treatment biomarkers and targets	Zhang et al. (2018d)
Esophageal cancer	Up	PTEN	*In vitro*, *In vivo*	EC-9706	Process of exosomal transfer and promote tumorigenic phenotype	A novel potential therapeutic approach	Ahmed et al. (2018)
	Up	PDCD4	*In vitro*	TE-1, Eca109/DDP, Het-1A	Significantly reduced the invasion ability	Affects the sensitivity of esophageal cancer to cisplatin	Lü et al. (2018)
	Up	PDCD4	Human, *In vitro*	EC9706	Promoted the migration and invasion	May become a potential biomarker for prognosis	Grassi et al. (2018)

PDCD4, Programmed Cell Death 4; PTEN, phosphatase and tensin homolog; Ras, Ras P21 Protein Activator; SMAD7, SMAD, Family Member 7; TPM1, Tropomyosin 1.

## Conclusion

Clinical and experimental data support the importance of MiR-21 in invasion, metastasis, cell growth, and apoptosis. MiR-21 acts as an oncogenic miRNA controlling downstream effectors linked to GI cancers. Solid and hematological cancers have a strong association with miR-21 overexpression. MiR-21 might be a biomarker for diagnosis and prognosis, as well as potential therapeutic target for many cancers of various kinds. Based on this hypothesis, new studies on miRNA signaling pathways have begun to examine how they differ from conventional protein signaling pathways. The function of miR-21 in anticancer drug resistance raises the possibility of using targeted therapeutic methods in addition to conventional cytotoxic drugs to inhibit miR-21 in a clinical setting to lessen cancer therapy resistance. Primary liver cancer patients, along with a few other solid tumor and hematological malignancies, are examined for safety following a miRNA-RX34 liposomal injection. We are unaware of any ongoing clinical trials involving miR-21 in cancer patients. Further study must be conducted prior to implementing miRNA-based cancer therapeutic methods in clinical settings. Also, the creation of efficient methods for delivering synthetic therapeutic miRNAs towards particular target tissues might boost the effectiveness of miRNA-mediated therapies, expanding the potential uses of this kind of therapy in the future for the treatment of cancer. While this review highlights the critical roles of miR-21 in gastrointestinal cancer pathogenesis, there are certain limitations that should be acknowledged. The studies referenced primarily focus on experimental models, and while the findings are compelling, their generalizability to broader patient populations remains uncertain. Furthermore, the lack of consistent clinical trial results involving miR-21, particularly in the context of therapeutic applications, highlights the need for more extensive research to better understand its potential as a diagnostic or therapeutic target. Future studies should address these limitations and explore the nuances of miR-21’s involvement in cancer biology across different stages and types of gastrointestinal cancers.
